# The E3 ligase MREL57 modulates microtubule stability and stomatal closure in response to ABA

**DOI:** 10.1038/s41467-021-22455-y

**Published:** 2021-04-12

**Authors:** Liru Dou, Kaikai He, Jialin Peng, Xiangfeng Wang, Tonglin Mao

**Affiliations:** State Key Laboratory of Plant Physiology and Biochemistry; Department of Plant Sciences, College of Biological Sciences, China Agricultural University, Beijing, China

**Keywords:** Plant sciences, Plant cytoskeleton, Proteolysis in plants, Drought

## Abstract

Regulation of stomatal movement is critical for plant adaptation to environmental stresses. The microtubule cytoskeleton undergoes disassembly, which is critical for stomatal closure in response to abscisic acid (ABA). However, the mechanism underlying this regulation largely remains unclear. Here we show that a ubiquitin-26S proteasome (UPS)-dependent pathway mediates microtubule disassembly and is required for ABA-induced stomatal closure. Moreover, we identify and characterize the ubiquitin E3 ligase MREL57 (MICROTUBULE-RELATED E3 LIGASE57) and the microtubule-stabilizing protein WDL7 (WAVE-DAMPENED2-LIKE7) in *Arabidopsis* and show that the MREL57-WDL7 module regulates microtubule disassembly to mediate stomatal closure in response to drought stress and ABA treatment. MREL57 interacts with, ubiquitinates and degrades WDL7, and this effect is clearly enhanced by ABA. ABA-induced stomatal closure and microtubule disassembly are significantly suppressed in *mrel57* mutants, and these phenotypes can be restored when *WDL7* expression is decreased. Our results unravel UPS-dependent mechanisms and the role of an MREL57-WDL7 module in microtubule disassembly and stomatal closure in response to drought stress and ABA.

## Abstract (web summary)

During stomatal opening and closing, the guard cell microtubule cytoskeleton is reorganised. Here the authors show that the E3 ubiquitin ligase MREL57 targets the microtubule stabilizing protein WDL7 to promote microtubule disassembly during ABA-induced stomatal closure.

## Introduction

Higher plants have developed finely tuned mechanisms to regulate gas exchange and water loss through stomata in leaves and stems. A pair of guard cells form stomatal pores and have developed intrinsic mechanisms to enable swelling or shrinkage, thus promoting the opening or closing of stomatal pores in response to environmental and stressful cues^1^. The stress-related plant hormone abscisic acid (ABA) is a key signal inducing stomatal closure in response to drought stress. Multiple studies addressing ABA-induced stomatal closure focus on ABA regulation of the activities of diverse membrane ion channels and transporters, such as H^+^, K^+^, and anion transporters and channels, through its receptor-phosphatase-kinase core signaling pathway, as well as the modulation of reactive oxygen species (ROS) and Ca^2+^ levels in the guard cells^2–4^. Other researchers have found that some ubiquitin E3 ligases participate in this physiological process. For example, a ubiquitin E3 ligase CHY ZINC-FINGER AND RING PROTEIN1 (CHYR1) promotes ABA-induced stomatal closure and its activity is regulated by Open Stomata 1 (OST1)/SnRK2.6-mediated protein phosphorylation^5^. However, how those E3 ligases function and their underlying mechanisms in ABA-induced stomatal closure are largely unclear.

The organization and dynamics of the microtubule cytoskeleton are altered in response to diverse developmental cues and external signals, such as phytohormones, light, and salt stress, and are thus critical for the adaptation of plants to environmental and stressful conditions^6–12^. Microtubule-associated proteins (MAPs) are the key regulators of microtubule dynamics, stability, and organization. Thus far, considerable progress has been made in understanding the molecular mechanism regarding how MAPs regulate microtubules to mediate plant cell growth in response to diverse signals^9^. For example, light signaling regulates the protein level of the MAP WAVE-DAMPENED2-LIKE3 (WDL3) to mediate hypocotyl cell elongation through the E3 ubiquitin ligase CONSTITUTIVE PHOTOMORPHOGENIC1 (COP1), which acts as a central repressor of seedling photomorphogenesis^7^. Although some studies with *Vicia faba* using the anti-microtubule drugs colchicine and paclitaxel suggested that microtubules have little impact on stomatal movement^13^; however, increasing evidence has produced conflicting results and support the notion that the microtubule cytoskeleton is vital for stomatal movement^14–17^. Pharmacological assays show that the microtubule-disrupting drug oryzalin inhibits stomatal opening, but that their closure is delayed by the microtubule-stabilizing drug paclitaxel^14,15^. Confocal imaging indicates that cortical microtubules undergo reorganizations during stomatal movement, including increasing assembly of cortical microtubules as stomata open and their disassembly as they close in response to ABA and darkness^15–17^. Although pharmacological evidence illustrates the critical impact of microtubules in stomatal movement, crucial genetic evidence is still lacking and the MAPs involved in stomatal movement are largely unidentified. In particular, much remains to be learned about the underlying regulatory mechanisms linking ABA and the MAP-microtubule modules and how they regulate stomatal closure in response to drought stress.

The ubiquitin-26S proteasome system (UPS) controls the degradation rates of numerous proteins and thus modulates multiple fundamental cellular and physiological processes, including plant growth, cell signaling, and stress responses^18,19^. The UPS consists of the concerted action of E1 Ub-activating enzymes, E2 Ub-conjugating enzymes, and E3 Ub ligases. The UPS leads to the covalent attachment of a multiubiquitin chain to target proteins, with the polyubiquitinated protein eventually being degraded by the 26S proteasome^19,20^. Increasing evidence shows that the UPS regulates the organization and dynamics of cortical microtubules during the plant cell response to diverse signals. For example, the microtubule-stabilizing protein SPIRAL1 is degraded by the UPS, which facilitates salt-induced microtubule disassembly and plant adaptation to high salinity conditions^21^. Khanna et al.^22^ showed that inhibition of proteolysis using the 26S proteasome inhibitor MG132 impairs ABA-induced microtubule disassembly and stomatal closure, which suggests that UPS-dependent MAP degradation is required for ABA-induced microtubule disassembly and stomatal closure. Thus, identification of the MAPs regulated by the UPS and participating in ABA-induced stomatal closure will boost our understanding of the mechanisms underlying ABA-regulated plant environmental adaptation.

In this study, we demonstrate that the UPS is crucial for ABA-induced microtubule disassembly and stomatal closure. Furthermore, we functionally characterize an E3 ligase-MAP (MREL57-WDL7) module having an important role in stomatal closure and microtubule depolymerization in response to drought stress and ABA treatment.

## Results

### UPS-mediated microtubule disassembly is required for ABA-induced stomatal closure

**Fig. 1:**
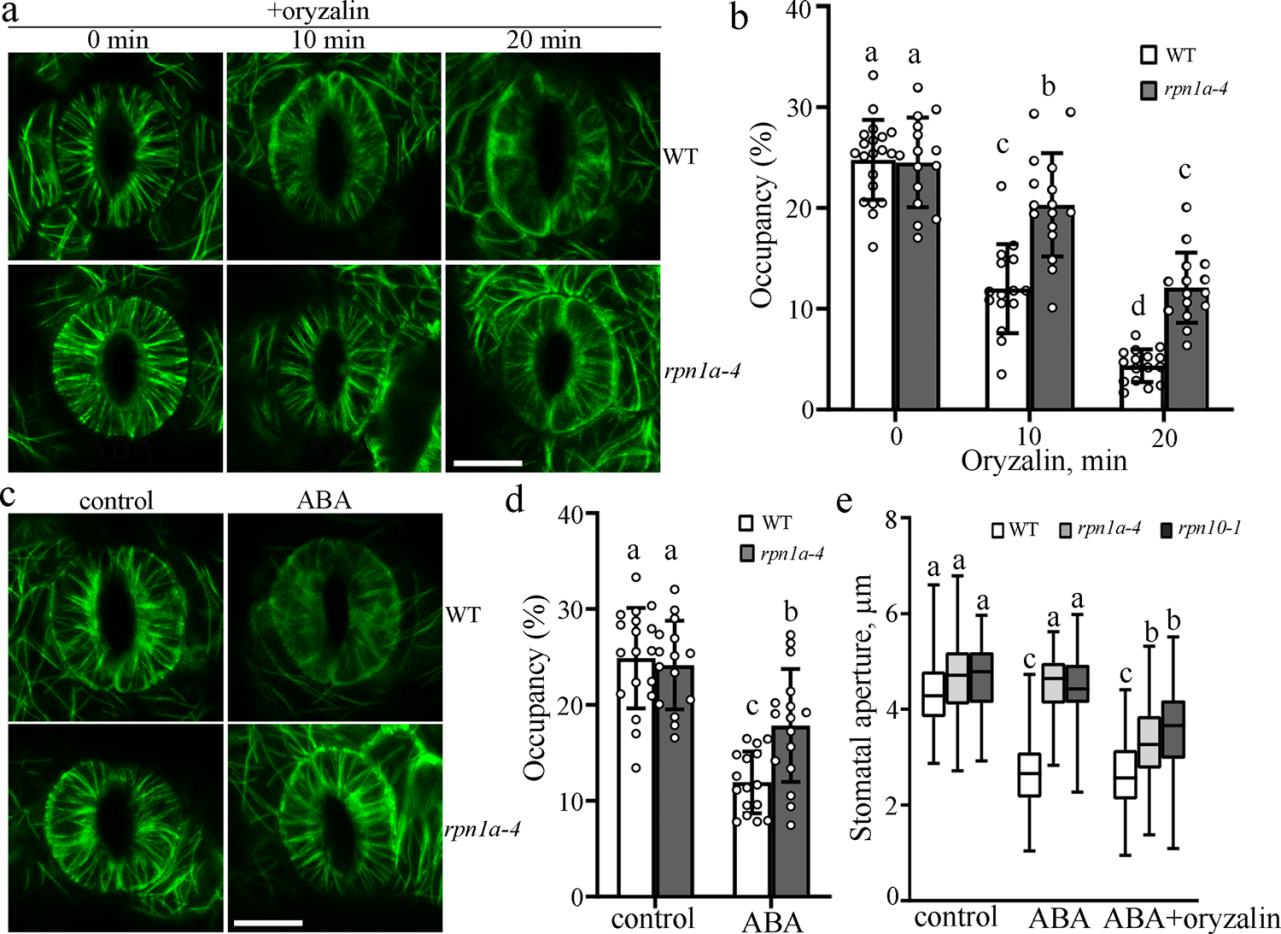
Cortical microtubules in 26S proteasome mutant plants are insensitive to ABA-induced disassembly. **a** Cortical microtubules in guard cells from WT and *rpn1a-4* seedlings treated with 5 μM oryzalin for the indicated times. Scale bar = 10 μm. **b** The graph shows the densities of microtubules in **a**. Data represent mean ± standard deviation (SD) values from three independent experiments with a minimum of 15 cells each. **c** Cortical microtubules in guard cells from WT and *rpn1a-4* seedlings treated with 10 μM ABA for 40 min. Scale bar = 10 μm. **d** The graph shows the densities of microtubules in **c**. Data represent mean ± standard deviation (SD) values from three independent experiments with a minimum of 15 cells each. **e** Detached rosette leaves from WT, *rpn1a-4*, and *rpn10-1* were incubated in opening buffer for 2 h and then treated with 10 μM ABA plus 0 or 5 μM oryzalin for 2 h. The box and whiskers plots represent minimum and maximum values. The line in the box indicates the median value and the boundaries demonstrate the 25th percentile (upper) and the 75th percentile (lower). Different letters represent significant differences at *p* < 0.01 (one-way ANOVA). The experiment was repeated three times as different biological replicates with a minimum of 100 stomatal pores.

A previous study showed that treatment with the 26S proteasome inhibitor MG132 suppressed ABA-induced microtubule disassembly and stomatal closure^22^, which led us to test whether the UPS has a role in regulating stomatal movement and microtubule stability in response to ABA. Thus, we chose 26S proteasome mutant *rpn1a-4* plants to investigate this hypothesis. Because ABA treatment results in the disassembly of cortical microtubules, we first determined microtubule stability in guard cells from *rpn1a-4* mutant using the microtubule-disrupting drug oryzalin, and microtubule density was quantified as described previously by Higaki et al.^23^. When the stomata were in an open state, the microtubules exhibited well-organized radial filaments in both wildtype (WT) and *rpn1a-4* guard cells. Although the density of cortical microtubules was clearly decreased in the WT guard cells after 10-min oryzalin treatment, it was slightly reduced in *rpn1a-4* guard cells. A longer duration resulted in the disappearance of most of the cortical microtubules in the guard cells in the WT but not *rpn1a-4* seedlings (Fig. 1a, b; Supplementary Fig. 1a), indicating that 26S proteasome-dependent proteolysis plays an important role in the regulation of microtubule stability in guard cells. In agreement with the results of oryzalin treatment, confocal imaging showed that cortical microtubules in guard cells from *rpn1a-4* seedlings were less sensitive to ABA-induced microtubule disassembly (Fig. 1c, d; Supplementary Fig. 1b). Phenotype analysis showed that the ABA-induced stomatal closure evident in the WT was significantly blocked in the *rpn1a-4* and *rpn10-1* (another 26S proteasome mutant) mutants. Importantly, this blockage was partially restored by the exogenous application of oryzalin (Fig. 1e). This evidence thus demonstrates that the UPS promotes microtubule disassembly, which is required for ABA-induced stomatal closure.

### MAP WDL7 protein is degraded in guard cells in response to ABA

**Fig. 2:**
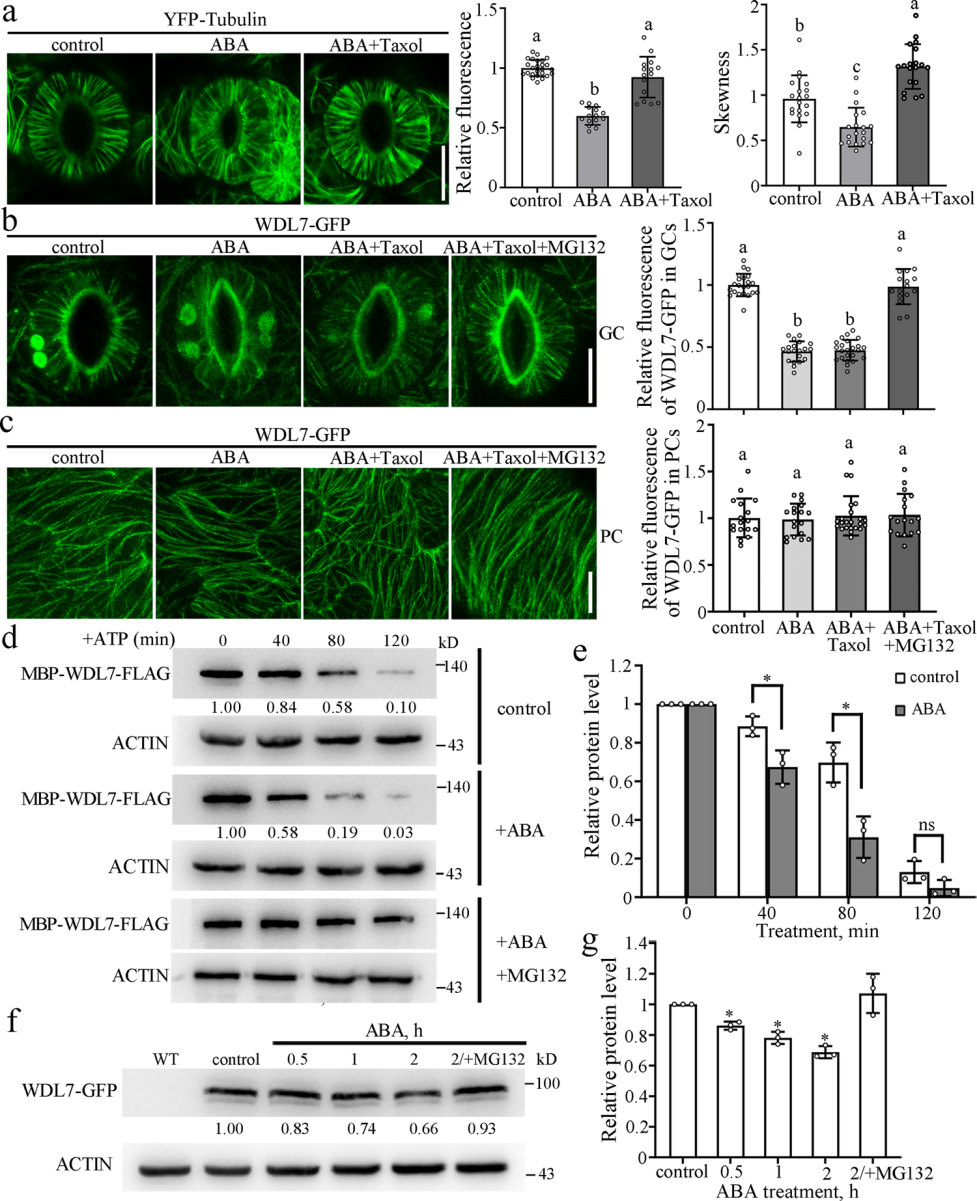
WDL7 is degraded during ABA-induced stomatal closure. **a** Detached rosette leaves from *YFP-tubulin* were incubated in opening buffer for 2 h and then treated with 10 μM ABA plus 0 or 20 μM Taxol for 40 min. Scale bar = 10 μm. The graphs show the relative fluorescence of YFP-tubulin and quantitative analysis of microtubule bundling (skewness). Data represent mean ± standard deviation (SD) values from three independent experiments with a minimum of 15 cells each. Different letters represent significant differences at *p* < 0.01 (one-way ANOVA). **b**, **c** Detached rosette leaves from *WDL7-GFP* transgenic seedlings were incubated in opening buffer for 2 h and then treated with 10 μM ABA, 10 μM ABA plus 20 μM Taxol, 10 μM ABA plus 20 μM Taxol and 50 μM MG132 for 40 min. Confocal images of the guard cells and pavement cells were taken. Scale bar = 10 μm. The graph shows the relative fluorescence of WDL7-GFP in **b** and **c**. Data represent mean ± standard deviation (SD) values from three independent experiments with a minimum of 15 cells each. Different letters represent significant differences at *p* < 0.01 (one-way ANOVA). **d** Ten-day-old WT seedlings were treated with mock buffer, 10 μM ABA, or 10 μM ABA plus 50 μM MG132 for 1 h and then total proteins were extracted. Purified MBP-WDL7-FLAG was incubated with equal amount of total proteins for the indicated times. MBP-WDL7-FLAG was detected with anti-FLAG antibody. Actin was used as a control. **e** Quantitative analysis of protein levels in **d**. The WDL7 protein level at 0 h was set to 1 as a reference for calculating the relative protein levels at the various time points. Data represent the mean ± SD for three independent experiments. Two-tailed Student’s *t* test, *p < 0.05. **f** Ten-day-old *WDL7-GFP* transgenic seedlings were treated with mock buffer, 10 μM ABA, or 10 μM ABA plus 50 μM MG132 for indicated times and then total proteins were extracted from the leaves. WDL7-GFP was detected with anti-GFP antibody. Actin was used as a control. **g** Quantitative analysis of protein levels in **f**. The protein level of WDL7-GFP treated with mock buffer was set to 1 as a reference for calculating relative protein levels of various time points. Data represent the mean ± SD for three independent experiments. Two-tailed Student’s *t* test, *p < 0.05.

Given the central role of UPS-promoted microtubule disassembly in the ABA-induced stomatal closure, identification and characterization of the MAPs involved in these physiological processes will facilitate our understanding of the underlying mechanisms of stomatal movement in the response of plants to diverse signals. We found that the total YFP-tubulin fluorescence was clearly decreased in guard cells after ABA treatment for 40 min. This decrease was owing to microtubule disassembly. Paclitaxel (Taxol) is a microtubule-stabilizing drug that promotes microtubule bundling and significantly suppresses microtubule disassembly^15,17,24,25^. We observed that the simultaneous treatment of guard cells with ABA and paclitaxel dramatically inhibited microtubule reduction and caused the microtubules to slightly bundle (Fig. 2a). Thus, we used this system to screen some members of WAVE-DAMPENED2 (WVD2)/WVD2-LIKE (WDL) family including WDL3, WDL4, WDL5, and WDL7, which we have focused on in recent years using GFP-tagged transgenic seedlings^7,8,26,27^. The total GFP signal was analyzed in the guard cells using an ABA stimulus as reference. We found that the total GFP fluorescence of a previously uncharacterized protein WDL7 was dramatically reduced in the guard cells from the WDL7-GFP transgenic seedlings following ABA treatment, even in the presence of paclitaxel. Furthermore, WDL7-GFP fluorescence was largely similar to the control condition in the guard cells when the 26S proteasome inhibitor MG132 was added into the system (Fig. 2b). In addition, WDL7-GFP protein levels were similar in the presence of oryzalin or paclitaxel alone (Supplementary Fig. 2). The GFP fluorescent spot color in Fig. 2b indicated autofluorescence emitted by chloroplasts (Supplementary Fig. 3). In particular, the total GFP fluorescence of WDL7 was not that different in the pavement or root epidermal cells after ABA treatment (Fig. 2c, Supplementary Fig. 4a–c), demonstrating that the decreased WDL7-GFP fluorescence in guard cells was not due to photobleaching. *WDL7-GFP* transgenic plants on a *mCherry-tubulin* background were further generated by crossing. In vivo time-course analysis showed that the microtubule disassembly preceded stomatal closure, and that degradation of WDL7-GFP temporally preceded microtubule disassembly and stomatal closure (Supplementary Fig. 5), implying that regulation of WDL7 protein level is critical for ABA-induced stomatal closure.

Then, we conducted cell-free degradation assays to confirm this degradation. Purified recombinant MBP-WDL7-FLAG protein from *Escherichia coli* was incubated with equal amounts of total protein extracted from ABA-treated and untreated WT seedlings in the presence of ATP. Immunoblot analysis showed that MBP-WDL7-FLAG was degraded after an extended time of ATP application in the WT without ABA treatment. Impressively, this degradation was significantly increased in the ABA-treated WT but was largely blocked by the addition of MG132 (Fig. 2d, e). Next, WDL7 protein levels were analyzed using WDL7-GFP transgenic seedlings. Western blotting using an anti-GFP antibody showed that the WDL7-GFP levels in the leaves were clearly decreased in the presence of ABA but largely restored when the seedlings were treated with both ABA and MG132 (Fig. 2f, g). These findings confirm that WDL7 is degraded through the 26S proteasome pathway during ABA-induced stomatal closure.

### Overexpression of *WDL7* renders stomatal closure less sensitive to ABA

**Fig. 3:**
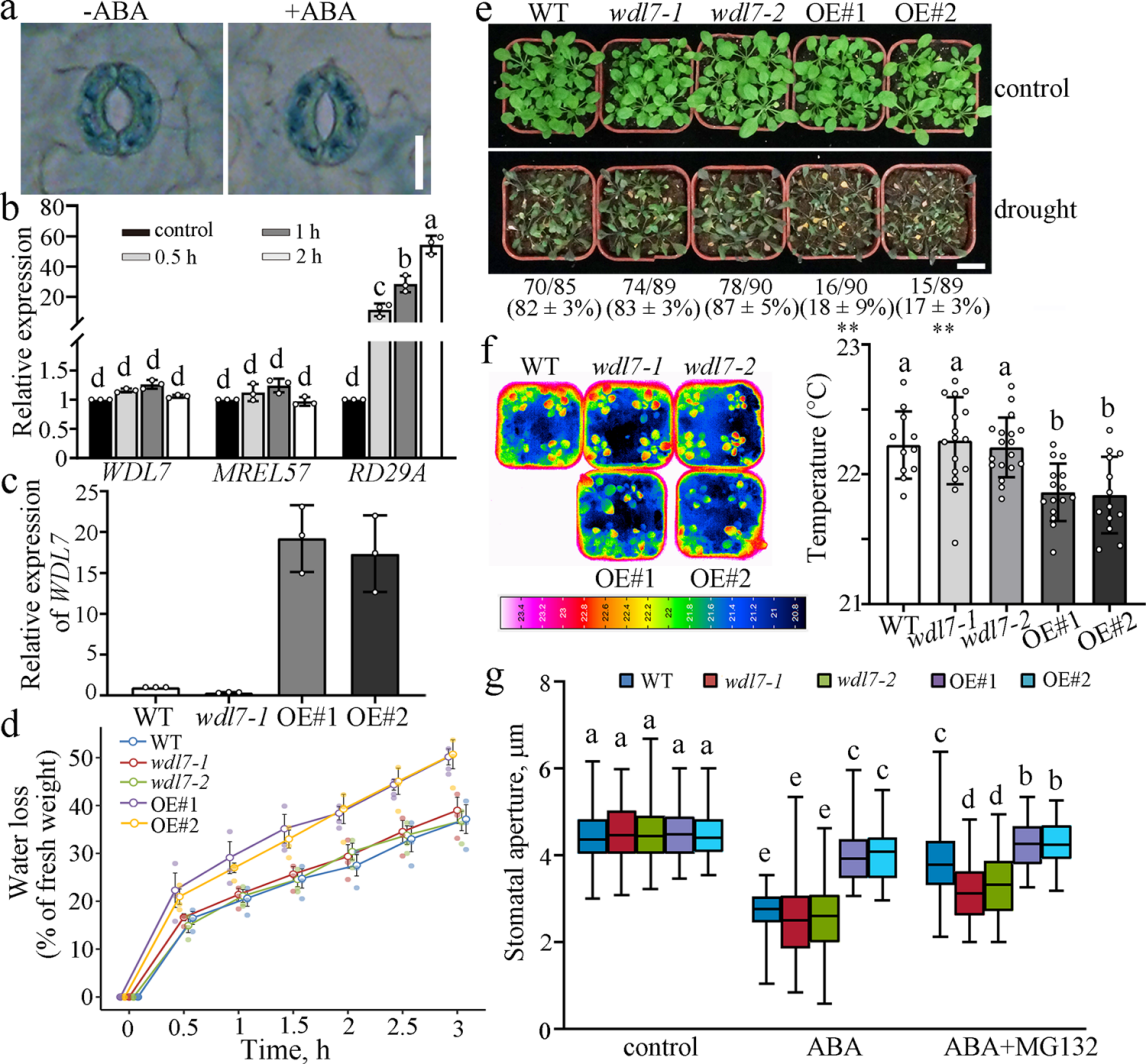
Overexpression of *WDL7* impairs the stomatal closure in response to ABA. **a** Detached rosette leaves from *Pro*_*WDL7*_*:GUS* transgenic seedlings were incubated in opening buffer for 2 h and then treated with or without 10 μM ABA for 2 h. The epidermal strips were peeled from the rosette leaves and GUS staining was performed. Scale bar = 10 μm. **b** Quantitative real-time PCR analysis of *WDL7* and *MREL57* RNA levels in leaves after various treatment durations using 10 μM ABA. *UBQ11* was used as a reference gene and *RD29A* was a positive control. Gene expression levels in seedlings treated with mock buffer were set to 1. The data represent the mean ± SD for three independent experiments. Significant differences from corresponding untreated seedlings are indicated by different letters (*p* < 0.01), as determined by one-way ANOVA. **c** *WDL7* expression was detected by quantitative real-time PCR in *wdl7-1* mutant and two *WDL7*-overexpressing lines (OE#1 and OE#2). The data represent the mean ± SD for three independent experiments. **d** Fresh weights of the detached leaves of seedlings from WT, *wdl7-1*, *wdl7-2*, OE#1, and OE#2 were measured at the indicated times. The experiment was repeated three times with independent treatments. Error bars indicate standard deviation. **e** Drought phenotypes of WT, *wdl7-1*, *wdl7-2*, OE#1, and OE#2 seedlings in soil. Three-week-old seedlings were subjected to drought stress by withholding water for two weeks before being photographed. Scale bar = 2 cm. Values represent mean ± SD for three independent experiments. Two-tailed Student’s *t* test, **p < 0.01. **f** Infrared thermography of seedlings from WT, *wdl7-1*, *wdl7-2*, OE#1, and OE#2. Images of 4-week-old plants in soil were taken using an infrared camera. The graph shows the leaf temperature measured using infrared camera software. Data represent mean ± standard deviation (SD) values. Different letters represent significant differences at *p* < 0.01 (one-way ANOVA). The experiment was repeated three times with independent treatments. **g** Detached rosette leaves of seedlings from WT, *wdl7-1*, *wdl7-2*, *OE#1*, and *OE#2* were incubated in opening buffer for 2 h and then treated with 10 μM ABA plus 0 or 50 μM MG132 for another 2 h. The box and whiskers plots represent minimum and maximum values. The line in the box indicates the median value and the boundaries demonstrate the 25th percentile (upper) and the 75th percentile (lower). Different letters represent significant differences at *p* < 0.01 (one-way ANOVA). The experiment was repeated three times as different biological replicates with a minimum of 100 stomatal pores.

To investigate the in vivo function of WDL7 in ABA-induced stomatal closure, the levels of the *WDL7* transcript were assessed using transgenic plants harboring β-glucuronidase (GUS) reporter gene under the control of the ~2-kb *WDL7* promoter. Twelve independent transgenic lines were stained for GUS activity. GUS staining showed that *WDL7* was widely expressed throughout the plant (Supplementary Fig. 6). Further analysis revealed that *WDL7* was expressed in stomata, but the staining was similar in the presence of ABA (Fig. 3a). RNA was purified from the leaves of WT seedlings treated with ABA, and quantitative real-time PCR analyses were performed. *WDL7* expression was largely similar after ABA treatment for 0, 0.5, 1, and 2 h (Fig. 3b, left panel). The ABA-responsive gene *RD29A* was used as a positive control (Fig. 3b, right panel). These results demonstrate that ABA does not affect the *WDL7* transcriptional level.

To dissect the physiological role of WDL7 in ABA-induced stomatal closure, we obtained a *wdl7-1* T-DNA insertion mutant and created another mutant (*wdl7-2*) using the CRISPR/Cas9 technique^28^ (Supplementary Fig. 7). In addition, of 15 WDL7-GFP-overexpressing lines obtained, lines 1 and 2 were selected for further analysis. The *WDL7* transcript level was dramatically reduced in the *wdl7-1* mutant and considerably increased in the overexpressing lines (OE#1 and OE#2, OE#1 was used in the previous assay of the WDL7-GFP signal) (Fig. 3c). A water loss assay using the detached rosette leaves of 4-week-old plants showed that the rate of water loss was similar in *wdl7* mutants and in WT but was faster in WDL7-GFP-overexpressing seedlings (OE#1 and OE#2) (Fig. 3d). We further compared their ability to avoid water loss by analyzing the difference in survival among all genotypes after soil drying. Consistent with the water loss results, there was no significant difference in the survival rates between the WT and *wdl7*-mutant seedlings. However, WDL7-GFP-overexpressing seedlings (OE#1 and OE#2) exhibited a hypersensitive phenotype with lower survival rates (Fig. 3e). In addition, an infrared camera was used to determine the transpiration rates of shoots by measuring the leaf temperature of 4-week-old plants. There was no significant difference in leaf temperature between *wdl7* mutants and WT. However, leaf temperatures were significantly lower in OE#1 and OE#2 than in WT (Fig. 3f), suggesting that WDL7-GFP-overexpressing leaves display a higher transpiration rate and lose more water than WT.

Furthermore, a stomatal aperture assay was performed to determine the function of WDL7 in ABA-induced stomatal closure. Stomatal closure was clearly delayed in OE#1 and OE#2, whereas *wdl7* mutants showed no significant difference from WT in response to ABA (Fig. 3g, left side). Among WVD2/WDL family members, WDL3 and WDL4 are closely related to WDL7^29^. GUS staining indicated that *WDL4* is not expressed in guard cells even after ABA treatment. *WDL3* is expressed in guard cells (Supplementary Fig. 8a). Stomatal closure in *wdl3-1* mutant was more sensitive to ABA treatment; however, WDL3-GFP protein was not degraded by this treatment (Supplementary Fig. 8b, c). These evidence suggest that the lack of a significant phenotype in the *wdl7* mutants in response to ABA is because ABA-induced stomatal closing involves the degradation of WDL7 protein. Accordingly, the 26S proteasome inhibitor MG132 partially repressed the sensitivity of stomatal closure to ABA in the WT seedlings, but the effect of MG132 was clearly inhibited in the *wdl7* mutants. For the WDL7-GFP-overexpressing seedlings (OE#1 and OE#2), the insensitivity of stomatal closure to ABA was enhanced when treated with both ABA and MG132 (Fig. 3g, right side). Taken together, these results demonstrated that WDL7 has a negative role in 26S proteasome degradation pathway-mediated stomatal closure, supporting the notion that WDL7 degradation via the 26S proteasome degradation pathway is essential for stomatal closure in response to drought stress and ABA treatment.

### WDL7 is a microtubule stabilizer

**Fig. 4:**
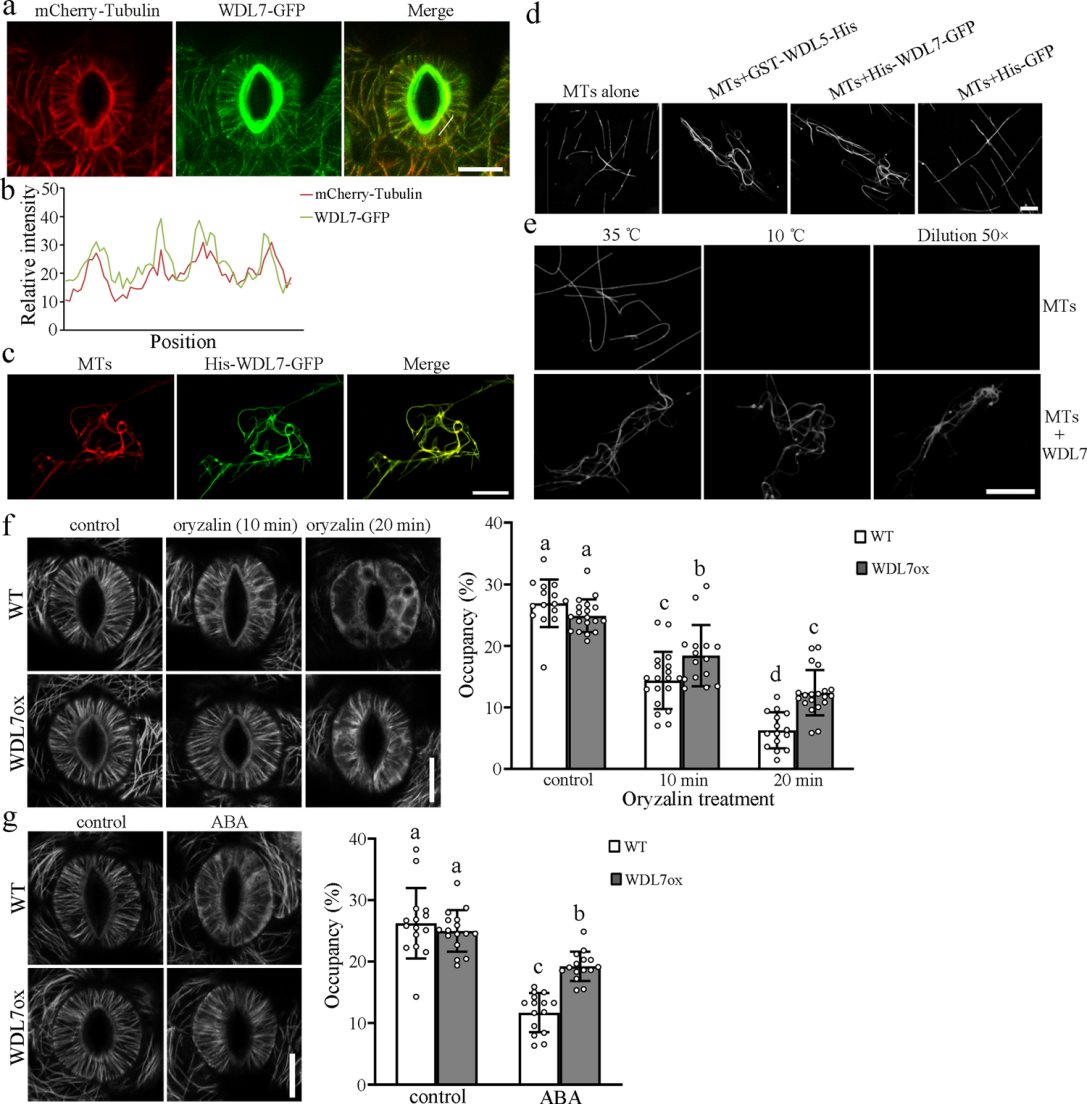
WDL7 binds to and stabilizes microtubules in vitro and in vivo. **a** WDL7-GFP colocalizes with cortical microtubules detected in guard cells. Detached rosette leaves of seedlings from *WDL7-GFP* transgenic plants on an *mCherry-tubulin* background were incubated in opening buffer for 2 h. The experiment repeated three times with similar results. Scale bar = 10 μm. **b** Plot of a line scan drawn in **a** showing a strong correlation between the spatial localization of WDL7-GFP and cortical microtubules. **c** His-WDL7-GFP colocalizes with microtubules polymerized from rhodamine-labeled tubulin in vitro. The experiment repeated three times with similar results. Scale bar = 10 μm. **d** WDL7 induced the formation of microtubule bundles in vitro. Images show microtubules polymerized from rhodamine-labeled tubulin incubated with 3 μM His-WDL7-GFP, GST-WDL5-His, or His-GFP protein for 30 min. The experiment repeated three times with similar results. Scale bar = 5 μm. **e** WDL7 stabilizes microtubules against cold and dilution-induced depolymerization. The experiment repeated three times with similar results. Scale bar = 10 μm. **f** Cortical microtubules in guard cells from WT and *WDL7-GFP* transgenic seedlings (WDL7ox) on an *mCherry-tubulin* background treated with 5 μM oryzalin for 10 and 20 min. Scale bar = 10 μm. The graph shows the densities of microtubules in **f**. Data represent mean ± standard deviation (SD) values from three independent experiments with a minimum of 15 cells each. **g** Cortical microtubules in guard cells from WT and *WDL7-GFP* transgenic seedlings (WDL7ox) on an *mCherry-tubulin* background treated with 0 and 10 μM ABA for 40 min. Scale bar = 10 μm. The graph shows the densities of microtubules in **g**. Data represent mean ± standard deviation (SD) values for three independent experiments with a minimum of 15 cells each. Different letters represent significant differences at *p* < 0.01 (one-way ANOVA).

Given that cortical microtubule destabilization is required for ABA-induced stomatal closure, we investigated the molecular basis for the WDL7 regulation of microtubules. We observed the subcellular localization of WDL7 in the guard cells using *WDL7-GFP* transgenic plants on an *mCherry-tubulin* background. Confocal microscopy showed that the WDL7-GFP fluorescence overlapped the red fluorescent signal of cortical microtubules (Fig. 4a), even in the presence of ABA, ABA plus Taxol, or MG132 (Supplementary Fig. 9a). Colocalization was analyzed by plotting the signal intensities of WDL7-GFP and microtubules using ImageJ software (Fig. 4b). The results demonstrated that WDL7 localized to microtubules in the guard cells. Moreover, confocal imaging showed that His-WDL7-GFP fusion protein directly bound to and bundled paclitaxel-stabilized microtubules that were polymerized from rhodamine-labeled tubulins (Fig. 4c, d), which is similar to other members from the WVD2/WDL family such as WDL3 and WDL5^26,27^.

The effects of WDL7 on microtubule stability were further investigated using cold and dilution treatments that can rapidly induce microtubule depolymerization^30^. Microtubules polymerized from rhodamine-labeled tubulin (20 μM) were incubated in the presence and absence of 3 μM His-WDL7-GFP (Fig. 4e, left panels). Then, the solutions were incubated at 10 °C for 30 min (Fig. 4e, middle panels) or diluted with 50× prewarmed buffer and incubated at 35 °C for 60 min (Fig. 4e, right panels). Confocal microscopy data showed that microtubule filaments were fully disassembled in the absence of WDL7, but that many microtubules persisted in the presence of WDL7 after the cold and dilution treatments. These results demonstrate that WDL7 can stabilize microtubules against low temperature- and dilution-induced depolymerization.

In order to characterize the effect of WDL7 on cortical microtubules in vivo, the microtubule-disrupting drug oryzalin was applied to guard cells against WT and WDL7-GFP-overexpressing seedlings. When the stomata were in an open state, the microtubules exhibited well-organized radial filaments in both the WT and WDL7-GFP-overexpressing guard cells. However, cortical microtubules were disrupted in the guard cells from WT after treatment with 5 μM oryzalin for 10 min, whereas the microtubules in WDL7-GFP-overexpressing guard cells were largely unaffected. Increases in the duration of oryzalin treatment resulted in disruption of the majority of cortical microtubules in WT but not WDL7-GFP-overexpressing guard cells (Fig. 4f; Supplementary Fig. 1c). Thus, microtubules were less sensitive to oryzalin treatment when the expression of *WDL7* was increased. In agreement with the above results, most of the cortical microtubules disappeared after ABA treatment in the WT guard cells, whereas many microtubules were observed in WDL7-GFP-overexpressing guard cells (Fig. 4g; Supplementary Fig. 1d). Previous studies showed that increased polymerization of the cortical microtubules is observed when stomata opening is induced by light^15^. We found that *WDL7* was expressed in guard cells in both the dark and light, and the light-induced stomatal opening was partially suppressed in the *wdl7* mutants (Supplementary Fig. 10a, b). Accordingly, microtubule assembly was partially suppressed in *wdl7*-mutant guard cells in response to light compared with WT (Supplementary Fig. 10c). These results confirm that WDL7 functions as a microtubule stabilizer.

### An E3 ligase MREL57 interacts with and ubiquitinates WDL7

**Fig. 5:**
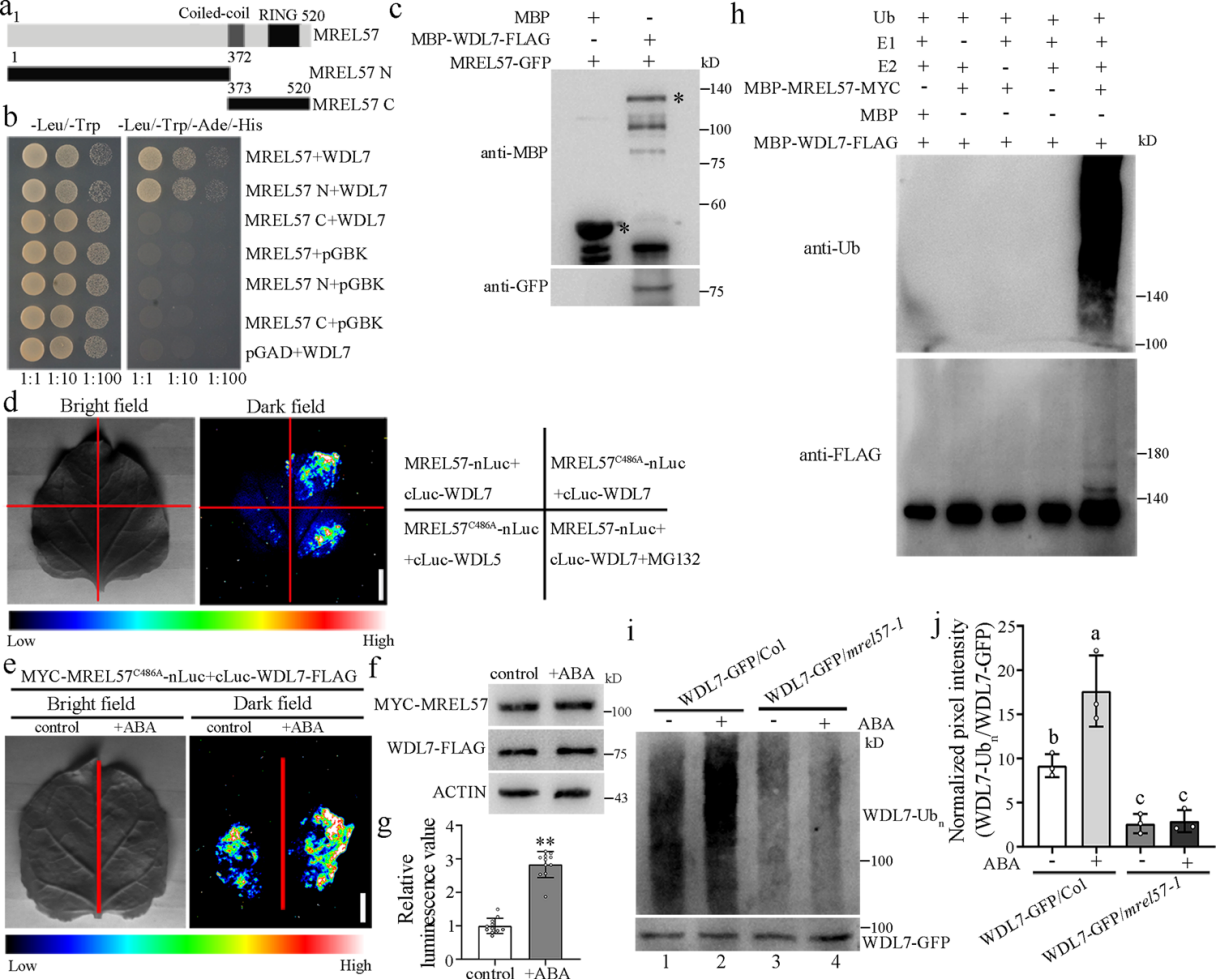
The E3 ligase MREL57 interacts with and ubiquitinates WDL7. **a** Schematic representation of MREL57 truncated proteins. **b** WDL7 interacts with MREL57 and MREL57 N in a yeast two-hybrid assay. **c** A semi-in vivo pull-down assay showed an interaction between MREL57 and WDL7. MBP-WDL7-FLAG and MBP were marked with asterisks, respectively. The experiment repeated three times with similar results. **d** Split-luciferase complementation assay to analyze the interaction between MREL57 and WDL7 in *N. benthamiana* leaves. MG132 at 50 μM was injected into the leaf tissues 12 h before imaging. The pseudo-color bar shows the range of luminescence. Scale bar = 1 cm. **e** ABA enhances the interaction between MREL57 and WDL7. *MYC*-*MREL57*^*C486A*^*-nluc* and *cluc-WDL7-FLAG* were transformed into *N. benthamiana* leaves. After a 2-day incubation, the leaves were treated with mock buffer or 100 μM ABA for 2 h. Scale bar = 1 cm. **f** WDL7 and MREL57 protein levels were detected by western blotting using anti-FLAG and anti-MYC antibodies. Actin was used as a control. **g** Luminescence values were determined using a spectrophotometer. The experiment was repeated three times. Data represent mean ± standard deviation (SD) values. Two-tailed Student’s *t* test, **p < 0.01. **h** MREL57 ubiquitinates WDL7 in vitro. Recombinant proteins were purified from *E. coli* and then incubated at 30 °C for 3 h. The ubiquitination of WDL7 was detected with anti-Ub and anti-FLAG antibodies. The experiment repeated three times with similar results. **i** MREL57 ubiquitinates WDL7 in vivo. WDL7-GFP and WDL7-GFP/*mrel57-1* transgenic seedlings were treated with 0 or 10 μM of ABA for 1 h in the presence of 50 μM MG132. Total proteins were extracted and incubated with anti-GFP mAb-magnetic agarose. Anti-Ub was used to detect the polyubiquitination of WDL7. **j** Quantitative analysis of the signal intensity in **i**. Data represent the mean ± SD for three independent experiments. Different letters represent significant differences at *p* < 0.01 (one-way ANOVA).

Given that WDL7 is degraded in response to ABA, we used WDL7 as the bait to screen for potential interacting proteins in a yeast two-hybrid screen. Six independent clones were isolated, which encoded deletion products of a potential E3 ligase. We named this gene *MREL57* (Microtubule-related E3 Ligase 57), which contains coiled-coil and RING domains in the C-terminal region (Fig. 5a). MREL57 is classified in cluster 2.3 according to cluster analysis and the metal-ligand arrangement of the RING domain^31^. The RING domain of MREL57 is highly conserved with well-characterized E3 ligases in cluster 2.3 (Supplementary Fig. 11a). An in vitro ubiquitination assay using an MBP-MREL57-MYC fusion protein showed that MREL57 was capable of autoubiquitination, as detected by anti-Ub and anti-MYC antibodies. When we mutated conserved amino acid in the RING-H2 finger domain (Cys-486 residue was replaced with Ala-486), which is responsible for binding to Zn^2+^, the MBP-MREL57^C486A^-MYC lost its E3 ligase activity (Supplementary Fig. 11b). These findings demonstrate that MREL57 is a new E3 ligase and that the RING-H2 domain is essential for its E3 ligase activity.

Our yeast two-hybrid assay data revealed that WDL7 interacted with full-length MREL57, but not with the known E3 ligases in cluster 2.3, including JUL1, MBR2, TEAR1, and TEAR2 (Fig. 5b, Supplementary Fig. 11c). In addition, MREL57 was unable to interact with either WDL3 or WDL4 in the yeast (Supplementary Fig. 11d). Previous studies showed that many members in WVD2/WDL family-like WDL3 and WDL5 participate in hypocotyl cell elongation^7,26,27^. Although *WDL7* was also expressed in the hypocotyls, no obvious hypocotyl-elongated phenotype of *wdl7* mutants was found in the light- and dark-grown seedlings (Supplementary Fig. 12). Thus, these results reveal the diverse physiological roles of WVD2/WDL proteins, and imply a specific MREL57-WDL7 module function in ABA-induced stomatal closure. Deletion experiments showed that WDL7 interacted with the MREL57 N terminus, but not with the C terminus (Fig. 5b). To further investigate the MREL57-WDL7 interaction, we generated MREL57-GFP transgenic plants driven by the *UBQ10* promoter and performed a semi-in vivo pull-down assay. The results showed that MBP-WDL7-FLAG, but not MBP, could successfully pull-down MREL57 in *Arabidopsis* (Fig. 5c). Furthermore, the in vivo interaction of WDL7 and MREL57 was validated by a firefly luciferase complementation imaging assay in tobacco leaves^32^. Although no reconstituted luminescence was detected when MREL57 and WDL7 were co-expressed in the absence of MG132, strong luminescence signals appeared in the presence of MG132 or when MREL57 was replaced with mutated MREL57 (MREl57^C486A^) (Fig. 5d). These results further confirmed that MREL57 interacts with and degrades WDL7. Moreover, we found that the interaction of MREL57 (using MREl57^C486A^) and WDL7 was significantly enhanced by ABA treatment (Fig. 5e–g).

The physical interaction between MREL57 and WDL7 prompted us to test whether WDL7 is a substrate of the MREL57 E3 ligase using an in vitro ubiquitination assay. MBP-WDL7-FLAG protein was purified from *E. coli* as substrate and MBP-MREL57-MYC recombinant protein acts as the E3 ligase. In the presence of E1, E2, and ubiquitin, MBP-WDL7-FLAG was polyubiquitinated by MBP-MREL57-MYC, as detected by anti-Ub and anti-FLAG antibodies (Fig. 5h). Furthermore, we examined the ubiquitination of WDL7 in the WDL7-GFP-overexpressing plants with or without ABA treatment. The polyubiquitination of WDL7 was detected with anti-Ub antibody, and the level of polyubiquitinated WDL7 was substantially increased by ABA treatment (Fig. 5i, Lanes 1 and 2; 5j, left panel). These results illustrate that WDL7 is a substrate of the MREL57 E3 ligase and that ABA enhances the MREL57 interaction with and ubiquitination of WDL7.

### MREL57 destabilizes microtubules and positively regulates stomatal closure in response to ABA

**Fig. 6:**
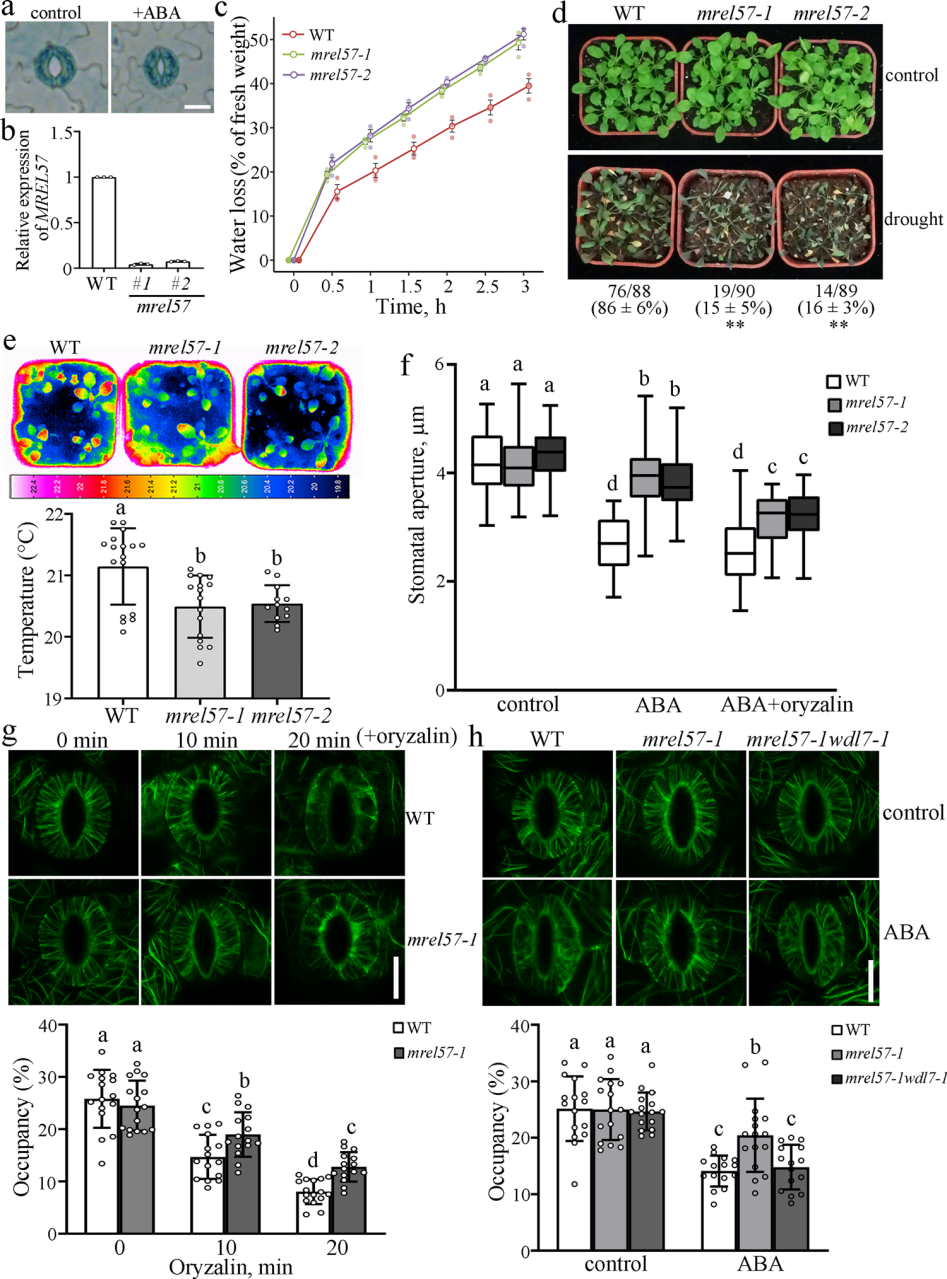
MREL57 destabilizes microtubules and positively regulates stomatal closure in response to ABA. **a** Detached rosette leaves from *Pro*_*MREL57*_*:GUS* transgenic seedlings were incubated in opening buffer for 2 h and then treated with or without 10 μM ABA for 2 h. The epidermal strips were peeled from the rosette leaves and GUS staining was performed. The experiment repeated three times with similar results. Scale bar = 10 μm. **b** *MREL57* expression was detected by quantitative real-time RT-PCR in WT, *mrel57-1*, and *mrel57-2* mutant seedlings. The experiment was repeated three times as different biological replicates. Error bars indicate standard deviation. **c** Fresh weights of detached leaves of seedlings from WT, *mrel57-1*, and *mrel57-2* were measured every 30 min for a total of 3 h. The experiment was repeated three times with independent treatments. **d** Drought phenotypes in the wildtype, *mrel57-1*, and *mrel57-2* in soil. Three-week-old seedlings were subjected to drought stress by withholding water for 2 weeks before being photographed. Values represent mean ± SD for three independent experiments. Two-tailed Student’s *t* test. **p < 0.01. **e** Infrared thermography of seedlings from WT, *mrel57-1*, and *mrel57-2*. Leaf temperature was measured using infrared camera software. The experiment was repeated three times with independent treatments. Error bars indicate standard deviation. Data represent mean ± standard deviation (SD) values. Different letters represent significant differences at *p* < 0.01 (one-way ANOVA). **f** Detached rosette leaves from WT, *mrel57-1*, and *mrel57-2* were incubated in opening buffer for 2 h and then treated with 10 μM ABA with or without oryzalin. The box and whiskers plots represent minimum and maximum values. The line in the box indicates the median value and the boundaries demonstrate the 25th percentile (upper) and the 75th percentile (lower). Different letters represent significant differences at *p* < 0.01 (one-way ANOVA). The experiment was repeated three times as different biological replicates with a minimum of 100 stomatal pores. **g** Cortical microtubules in guard cells from WT and *mrel57-1* seedlings treated with 5 μM oryzalin for the indicated times. Scale bar = 10 μm. The graph shows the densities of microtubules. Data represent mean ± standard deviation (SD) values from three independent experiments with a minimum of 15 cells each. **h** Cortical microtubules in guard cells from WT, *mrel57-1* mutant, and *mrel57-1wdl7-1* double mutant seedlings treated with 10 μM ABA for 40 min. Scale bar = 10 μm. The graph shows the densities of microtubules. Data represent mean ± standard deviation (SD) values from three independent experiments with a minimum of 15 cells each.

Given that MREL57 interacts with and ubiquitinates WDL7, we proposed that MREL57 has a role in regulating microtubule stability and stomatal closure in response to drought stress and ABA treatment. GUS staining of transgenic seedlings carrying *Pro*_*MREL57*_:GUS indicated that *MREL57* was constitutively expressed in most tissues (Supplementary Fig. 13). Furthermore, *MREL57* was expressed in stomata, with no clear difference after ABA treatment (Fig. 6a). Real-time PCR further confirmed that *MREL57* expression was not regulated by ABA treatment (Fig. 3b, middle panel). We obtained two T-DNA insertion mutants (*mrel57-1* and *mrel57-2*) for *MREL57*. Real-time qPCR analysis determined that the transcript accumulation of *MREL57* was significantly decreased in *mrel57-1* and *mrel57-2* mutants (Fig. 6b). A water loss assay showed that the water loss rate of detached leaves was faster in *mrel57* mutants than in WT (Fig. 6c). Accordingly, the *mrel57* mutant seedlings were more sensitive to drought stress and leaf temperatures were significantly lower in *mrel57* mutants than in WT (Fig. 6d, e), suggesting that *mrel57* mutant leaves display a higher transpiration rate and lose more water than the WT. The stomatal aperture assay showed that stomatal closure was less sensitive to ABA treatment in *mrel57* mutants compared with WT (Fig. 6f, left and middle panels). This evidence demonstrates that MREL57 participates in promoting stomatal closure in response to drought stress and ABA treatment.

Next, we further investigated how MREL57 regulates microtubule stability during ABA-induced stomatal closure. We found that disruption of microtubules with oryzalin in the *mrel57* mutant partially restored stomatal closure under ABA treatment (Fig. 6f, right panel), suggesting that microtubules are more stable in *mrel57* guard cells. To test this hypothesis, we crossed *mrel57-1* mutant with *YFP-tubulin* plants. Confocal microscopy showed that the microtubules exhibited well-organized radial filaments in both the WT and *mrel57-1* guard cells on a *YFP-tubulin* background when the stomata were in an open state. After oryzalin treatment for 10 min, many microtubules were disrupted in the WT guard cells, but not in the *mrel57-1* guard cells. After a longer treatment, most of the microtubules disappeared in WT guard cells, but more microtubules were observed in *mrel57-1* guard cells (Fig. 6g, Supplementary Fig. 1e). In agreement with the results of oryzalin treatment, microtubules mostly disassembled in the WT guard cells but persisted in the *mrel57-1* guard cells after ABA treatment for 40 min (Fig. 6h, left panel, Supplementary Fig. 1f). Taken together, these results demonstrate that MREL57 promotes microtubule disassembly to mediate ABA-induced stomatal closure.

In addition, the water loss, leaf temperature, and stomatal closure phenotypes of *mrel57* mutants were fully rescued by *MREL57-GFP* (Com#1 and Com#2), but not by mutated *MREL57*^*C486A*^*-GFP* (Com^C486A^#1 and Com^C486A^#2) driven by the native *MREL57* promoter (Supplementary Fig. 14), demonstrating that the phenotype is indeed caused by the loss of MREL57 function and that the function of MREL57 depends on its E3 ligase activity. These results support the notion that MREL57 is required for ABA-induced stomatal closure by destabilizing microtubules.

### MREL57 is responsible for WDL7 degradation in ABA-induced stomatal closure and microtubule disassembly

**Fig. 7:**
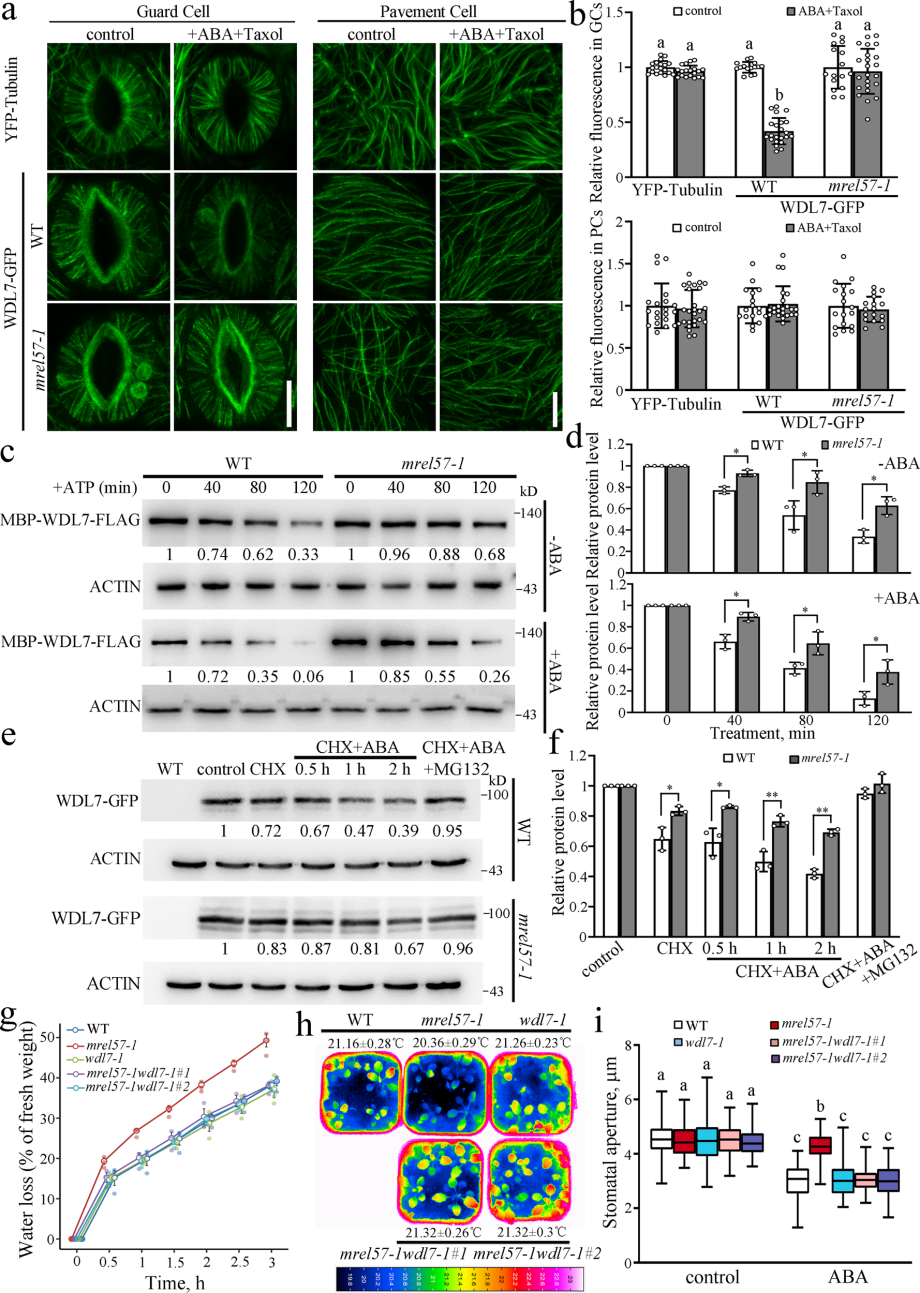
Degradation of WDL7 is required for MREL57-mediated stomatal closure in response to ABA. **a** Detached rosette leaves of seedlings from *YFP-tubulin*, *WDL7-GFP*, and *WDL7-GFP*/*mrel57-1* were incubated in opening buffer for 2 h and then treated with 10 μM ABA plus 20 μM Taxol for 40 min. Confocal images of the guard cells and pavement cells were taken. Scale bar = 10 μm. **b** The graphs show the relative fluorescence in **a**. Data represent mean ± standard deviation (SD) values from three independent experiments with a minimum of 15 cells each. Different letters represent significant differences at *p* < 0.01 (one-way ANOVA). **c** Degradation of WDL7 was clearly decreased in *mrel57-1* mutant in a cell-free degradation assay. Purified MBP-WDL7-FLAG was incubated with equal amounts of total proteins from 10-day-old WT and *mrel57-1* seedlings. MBP-WDL7-FLAG was detected with anti-FLAG antibody. Actin was used as a control. **d** Quantitative analysis of protein levels in **c**. The WDL7 protein level at 0 h was set to 1 as a reference for calculating the relative protein levels at the various time points. Data represent the mean ± SD for three independent experiments. Two-tailed Student’s *t* test, *p < 0.05. **e** Ten-day-old *WDL7-GFP* and *WDL7-GFP/mrel57-1* transgenic seedlings were treated with 100 μM CHX, 100 μM CHX plus ABA, or 100 μM CHX plus ABA and MG132 for the indicated times and then total protein was extracted from the leaves. WDL7-GFP was detected with an anti-GFP antibody. Actin was used as a control. **f** Quantitative analysis of the protein levels in **e**. The WDL7 protein level in the leaves from mock buffer-treated seedlings was set to 1 as a reference for calculating the relative protein levels at the various time points. Data represent the mean ± SD for three independent experiments. Two-tailed Student’s *t* test, *p < 0.05, **p < 0.01. **g**, Fresh weights of detached leaves of seedlings from WT, *mrel57-1*, *wdl7-1*, *mrel57-1wdl7-1#1*, and *mrel57-1wdl7-1#2* were measured every 30 min over a total of 3 h. The experiment was repeated three times with independent treatments. Error bars indicate standard deviation. **h** Infrared thermography of seedlings from WT, *mrel57-1*, *wdl7-1*, *mrel57-1wdl7-1#1*, and *mrel57-1wdl7-1#2*. Leaf temperature was measured using infrared camera software. Data represent mean ± standard deviation (SD) values. **i** Detached rosette leaves of seedlings from WT, *mrel57-1*, *wdl7-1*, *mrel57-1wdl7-1#1*, and *mrel57-1wdl7-1#2* were incubated in opening buffer for 2 h and then treated with 10 μM ABA for 2 h. The box and whiskers plots represent minimum and maximum values. The line in the box indicates the median value and the boundaries demonstrate the 25th percentile (upper) and the 75th percentile (lower). Different letters represent significant differences at *p* < 0.01 (one-way ANOVA). The experiment was repeated three times as different biological replicates with a minimum of 100 stomatal pores.

MREL57 interacts with the microtubule-stabilizing protein WDL7 and destabilizes microtubules to promote stomatal closure under ABA treatment, which led us to investigate whether MREL57 mediates the degradation of WDL7 during ABA-induced stomatal closure. We crossed the WDL7-GFP transgenic plant with *mrel57-1* mutant to examine the protein level of WDL7-GFP on an *mrel57-1* mutant background. Paclitaxel was used to stabilize microtubules under ABA treatment. When the stomata were in an open state, WDL7-GFP exhibited well-organized radial filaments in both WDL7-GFP-overexpressing WT and *mrel57-1* guard cells. After ABA plus paclitaxel treatment for 40 min, the total WDL7-GFP fluorescence was dramatically reduced in the guard cells but not in pavement cells from the WDL7-GFP-overexpressing seedlings. However, WDL7-GFP signal remained largely unaffected in WDL7-GFP/*mrel57-1* seedlings (Fig. 7a, b). In addition, most of the WDL7-GFP colocalized to the microtubules in the *mrel57* mutant guard cells in the presence of ABA and paclitaxel (Supplementary Fig. 9b). These results suggested that MREL57 mediates WDL7 degradation during ABA-induced stomatal closure.

Next, we conducted a cell-free degradation assay. Purified recombinant MBP-WDL7-FLAG protein was incubated with equal amounts of total protein extracted from WT and *mrel57-1* seedlings in the presence of ATP. Immunoblot analysis showed that MBP-WDL7-FLAG was degraded after an extended period of ATP application in both the WT and *mrel57-1* mutant; however, this degradation was much slower in the *mrel57-1* mutant, even when these seedlings were treated with ABA (Fig. 7c, d). WDL7-GFP transgenic WT and *mrel57-1* seedlings were then treated with 100 μM cycloheximide (CHX), a protein synthesis inhibitor, to further examine the effect of MREL57 on WDL7 degradation in response to ABA in vivo. We found that, although MREL57 could coimmunoprecipitate with WDL7 in both the leaves and roots, WDL7-GFP protein levels were only decreased in the leaves after ABA treatment (Fig. 2f, Supplementary Fig. 4d, e), suggesting that the MREL57-WDL7 module might mainly function in the leaves in response to ABA treatment. Accordingly, western blotting showed that the degradation of WDL7-GFP in leaves was clearly reduced in the *mrel57-1* mutant compared with the WT, and this degradation was largely suppressed by MG132 (Fig. 7e, f). Consistent with this result, ABA-induced WDL7 ubiquitination was also significantly reduced in the *mrel57-1* mutant (Fig. 5i, Lanes 3 and 4; 5j, right panel). Based on these combined results, we conclude that MREL57 is responsible for WDL7 degradation during ABA-induced stomatal closure.

To elucidate the genetic interactions of MREL57 and WDL7, we generated *mrel57-1wdl7-1* double mutants (*mrel57-1wdl7-1#1* and *mrel57-1wdl7-1#2*) by crossing. A water loss assay showed faster water loss in detached leaves of *mrel57-1* mutant, but not in those of *mrel57-1wdl7-1* double mutants, which exhibited a similar water loss phenotype to WT and *wdl7-1* mutant (Fig. 7g). Similarly, there was no significant difference in leaf temperature between *mrel57-1wdl7-1* double mutants from WT and *wdl7-1* mutant (Fig. 7h). Importantly, the insensitivity of ABA-induced stomatal closure and microtubule disassembly phenotypes in *mrel57-1* mutant were clearly suppressed in *mrel57-1wdl7-1* double mutants (Fig. 6h, right panels; 7i). Taken together, these results support the idea that MREL57-mediated WDL7 degradation is essential for microtubule disassembly and stomatal closure in response to drought stress and ABA treatment.

## Discussion

**Fig. 8:**
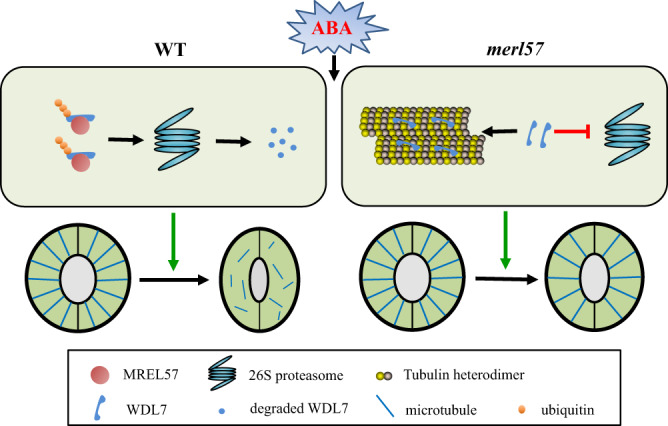
A hypothetical model for MREL57-WDL7 module function in stomatal closure in response to ABA. Working model for the role of WDL7 proteolysis in ABA-induced stomatal closure and microtubule disassembly in WT and *mrel57* mutant guard cells. WDL7 is a microtubule-stabilizing protein. In WT, the E3 ligase MREL57 ubiquitinates and degrades WDL7 in response to ABA, which facilitates microtubule disassembly and stomatal closure. In *mrel57* mutant, the degradation of WDL7 is blocked. WDL7 binds to and stabilizes microtubules, resulting in stomatal closure insensitivity to ABA in *mrel57* mutant.

In this study, we show that the UPS-mediated stability of cortical microtubules is critical for ABA-induced stomatal closure. Furthermore, MREL57-WDL7 module was identified to participate in microtubule disassembly and stomatal closure in response to drought stress and ABA treatment. Our results shed light on microtubule reorganization in guard cells in response to ABA and suggest a hypothetical model for MREL57-WDL7 function in ABA-induced stomatal closure (Fig. 8).

Cortical microtubules in guard cells undergo dynamic reorganization during stomatal movement. We found that microtubule disassembly preceded stomatal closure, which behavior was similar to that observed with microtubule reorganization and hypocotyl elongation in response to light and hormones^11^, supporting the notion that microtubule regulation facilitates stomatal movements. Microtubules are destabilized when stomata closure is induced by ABA and dark conditions, and exogenous application of the microtubule-stabilizing drug paclitaxel partially blocks stomatal closure, suggesting that the regulation of cortical microtubule stability is important for proper stomatal closure^15,16^. In this study, we provided several lines of evidence supporting the hypothesis that cortical microtubule destabilization is required for ABA-induced stomata closure. Cortical microtubules are more stable in *rpn1a-4* and *mrel57* guard cells, and the microtubule-disrupting drug oryzalin partially restores the lower sensitivity of ABA-induced *rpn1a-4* and *mrel57* stomatal closures. WDL7, as a microtubule-stabilizing protein, has a negative role in UPS-regulated ABA-mediated microtubule disassembly and stomatal closure. Lower *WDL7* expression clearly restored the lower sensitivity of *mrel57* mutant to ABA-induced microtubule disassembly and stomatal closure. Our findings are in agreement with previous studies showing that ABA-induced stomatal closure and cortical microtubule destabilization were impaired in the E3 ligase *CONSTITUTIVE PHOTOMORPHOGENIC 1* (*COP1*) mutant^22^. Besides WDL7, the MAPs WDL3 and SPR1 also function as microtubule-stabilizing proteins and are regulated by the UPS^9^. Taken together, these studies, therefore, suggested that the UPS mainly induces microtubule destabilization through degradation of diverse microtubule-stabilizing proteins, which is required for multiple UPS-mediated physiological processes, including ABA-induced stomatal closure. Future studies will provide more experimental evidence to address the characteristics of the UPS in microtubules in the response of plants to other developmental and environmental cues.

Microtubules provide the trajectories for cellulose synthase complexes in the plasma membrane and guide the deposition direction of cellulose microfibrils to support plant cell growth. This mechanism has been well investigated in both roots and hypocotyls^33,34^. Cellulose also undergoes significant reorganization during stomatal movement from a relatively uniform distribution in the open state to bundles in the closed state^35^. However, the relationship between cellulose reorganization and cortical microtubules during stomatal movement is still unclear. We found that the average angles of the microtubules, which are relative to the stomata inner surface, were reduced in WT but not in *rpn1a-4* mutant, *WDL7-*overexpressing, or *mrel57-1* mutant guard cells after ABA treatment (Supplementary Fig. 15). Whether microtubule reorganization affects the arrangement of cellulose, which facilitates repetitive cell wall expansion and contraction during stomatal movement, will be an interesting question to be answered in future work.

Our study indicated that WDL7 has a negative role in UPS-mediated ABA-regulated stomatal closure. We did not observe an obvious phenotype for ABA-induced stomatal closure in *wdl7* mutants. Although WDL3, a close homolog of WDL7 in WVD2/WDL family, is involved in ABA-induced stomatal closure, their regulatory mechanisms are quite different, suggesting that the lack of a stomatal closure phenotype in the *wdl7* mutants maybe not due to redundancy between WDL7 and other WVD2/WDL family members but due to the degradation of WDL7 protein in response to ABA. This phenomenon has been well reported in some physiological processes involving E3 ligases. For example, COLD-REGULATED GENE 27 (COR27) and COR28 are negative regulators of photomorphogenesis, and they undergo COP1-mediated degradation to avoid too long hypocotyls in darkness. Thus, dark-grown *cor27 cor28* double mutant seedlings exhibited no hypocotyl-elongated phenotype owing to the degradation of COR27 and COR28^36^. Our study showed that WDL7 degradation facilitated microtubule disassembly and ABA-induced stomatal closure. Further work is necessary to determine if WDL7 is required in general for stomatal movement, including in response to other environmental cues, such as changes in atmospheric CO_2_, and temperature.

In this study, we showed that *MREL57* and *WDL7* transcripts are not regulated by ABA but that the interaction and ubiquitination of MREL57 with WDL7 were clearly enhanced by ABA, suggesting that ABA is critical for regulating the activities of MREL57. This phenomenon has been broadly studied in multiple physiological processes involving E3 ligases. For example, SnRK2.6/OST1 kinase phosphorylates and enhances E3 ligase CHYR1 activity during ABA-induced stomatal closure^5^. We found that WDL7 degradation was significantly suppressed in *abscisic acid insensitive 1-1* (*abi1-1*) mutants (Supplementary Fig. 16a). Bioinformatics analysis further showed the presence of some potential OST1 phosphorylation motifs in the amino-acid sequence of MREL57 (http://phosphat.uni-hohenheim.de/phosphat.html?code=AT5G24870.1). Moreover, we found that OST1 interacts with MREL57, and WDL7 degradation was much slower in the *ost1-3* mutants (Supplementary Fig. 16b, c). These findings suggest that ABA might regulate the function of the MREL57-WDL7 module through its signaling pathway. Our data indicated that the MREL57-WDL7 module mainly functions in the leaves in response to ABA treatment. Previous studies showed that mesophyll ABA restrains early growth and flowing under non-stress conditions, allowing plants to accumulate biomass and maximize yield^37^. Moreover, mesophyll cells produce significant amounts of ABA under water deficit^38^. Our western blotting results suggested that MREL57 might also interact with and degrade WDL7 in mesophyll cells; however, the role and detailed mechanisms of the MREL57-WDL7 module with ABA treatment in this cell type requires further investigation. In this study, we obtained strong evidence that the MREL57-WDL7 module functions in ABA-induced stomatal closure. These findings provide a new perspective on the cellular control of stomatal movement and further our understanding of the molecular basis for the effects of ABA on the responses of plants to environmental and stressful conditions.

## Methods

### Plant materials and growth conditions

The WT ecotype used in this study was *A. thaliana* Columbia (Col) and all plant materials were from the Col background. Seeds were sown on 1/2 MS medium (Sigma-Aldrich) with 0.8% agar and 1% sucrose (w/v). The 10-day-old seedlings were transferred into soil and were grown under a 16-h/8-h light/dark photoperiod in a growth room at 22 °C. *35S: YFP-tubulin5A* transgenic plants, *35S: mCherry-tubulin5A* transgenic plants, *35S: WDL3-GFP* transgenic plants, *Pro*_*WDL3*_: GUS transgenic plants, *rpn1a-4*, *rpn10-1*, *abi1-1*, *ost1-3*, *wdl3-1*, and *wdl5-1* were reported previously^7,21,26,27,39–42^. T-DNA insertion mutants, *wdl7-1* (SALK_081293), *mrel57-1* (SALK_020143), and *mrel57-2* (SALK_015310), were obtained from the *Arabidopsis* Biological Resource Center. For overexpressing transgenic plants, the coding sequences of *WDL7* and *MREL57* were inserted into *pCAMBIA1390* and *pBI121* vectors under the *UBQ10* promoter. To generate *Pro*_*WDL4*_:GUS, *Pro*_*WDL7*_:GUS, and *Pro*_*MREL57*_:GUS transgenic plants, the promoters of *WDL4*, *WDL7* and *MREL57* were inserted into *pCAMBIA1391* vector. To complement the phenotype of the *mrel57* mutant, the coding sequence and the promoter of *MREL57* were amplified and reconstructed into a *pCAMBIA1300* vector. Mutant *wdl7-2* was generated by an egg cell-specific promoter-controlled CRISPR/Cas9 system^28^. Constructs were transformed into *Arabidopsi*s plants by Agrobacterium (strain GV3101). Homozygous lines were used for subsequent analyses. The *35S: YFP-tubulin5A/rpn1a-4*, *35S:YFP-tubulin5A/mrel57-1*, *35S:YFP-tubulin5A/wdl7-1*, *35S:YFP-tubulin5A/mrel57-1wdl7-1*, *UBQ:WDL7-GFP/mrel57-1*, *UBQ*:*WDL7-GFP*/*ost1-3*, *mrel57-1wdl7-1*, *UBQ: WDL7-FLAG MREL57-GFP*, *UBQ:WDL7-GFP mCherry-tubulin*, *UBQ:WDL7-GFP mCherry-tubulin*/*mrel57-1* plants were generated by crossing. Primers are listed in Supplementary Table 1.

### Cell-free protein degradation assay

A cell-free protein degradation assay was performed as described previously^43^. Total WT, *mrel57* or *abi1-1* mutant proteins were extracted with degradation buffer (25 mM Tris-HCl, pH 7.5; 10 mM NaCl; 10 mM MgCl_2_; 4 mM phenylmethylsulfonyl fluoride; 5 mM DTT; 10 mM ATP). Purified MBP-WDL7-FLAG protein (100 ng) was incubated with 500 μg total proteins at 23 °C for the indicated time. The products were subjected to western blotting with anti-FLAG antibody (ABclonal AE005, 1/5000) and anti-actin antibody (ABclonal AC009, 1/5000).

### Water loss assay and drought treatment

For a water loss assay with detached leaves, rosette leaves were cut from 4-week-old plants in soil. The weight of the detached leaves was measured every 0.5 h over ~3 h. For the drought treatment, 7-d-old seedlings were transferred to pots containing the same amount of soil for 2 weeks under normal short-day conditions. After each pot absorbed water fully, the plants were subjected to drought conditions by withholding water for 2 weeks^44,45^. All of the pots for each genotype were distributed in the flat randomly and were rotated frequently to minimize the influence from the environment^46^. Plants that maintained well-watered conditions were used as a control.

### Stomatal aperture assay

Stomatal aperture assays were performed as previously described^45,47^. To examine ABA-induced stomatal closure, the rosette leaves of 4-week-old plants in soil were incubated in opening solution (2-(N-morpholino)ethanesulfonic acid; MES buffer: 50 mM KCl, 10 mM CaCl_2_, and 10 mM MES, pH 6.15) in a growth chamber for 2 h to completely open the stomata. Then, the rosette leaves were transferred to opening buffer containing 0 and 10 μM ABA for 2 h. To examine light-induced stomatal opening, the rosette leaves of 4-week-old plants in soil were incubated in MES buffer in a dark growth chamber for 4 h to close the stomata. Then, the rosette leaves were transferred to the light condition for 1 h. The epidermal strips were peeled from rosette leaves and photographed with an OLYMPUS BX51 microscope. Stomatal apertures were measured with ImageJ software.

### Infrared thermograph imaging

Thermal imaging was used to monitor leaf temperature as described previously^48^. Four-week-old plants in soil were transferred from high humidity (RH 70%) to low humidity (RH 40%) growth conditions. The plants were photographed with a thermal imaging camera (VarioCAM^®^ HD), and the leaf temperatures measured using InfraTec IRBIS 3 software (http://www.InfraTec.net/). Plants in three different pots were analyzed for each genotype, with similar results.

### Histochemical GUS activity analysis

For expression patterns of *WDL7* or *MREL57*, seedlings grown in 1/2 MS or soil were subjected to GUS staining. To test the histochemical localization of GUS activity in guard cells, detached rosette leaves from 4-week-old plants in soil were used. The epidermal strips were peeled from the rosette leaves and GUS staining was performed. To examine the histochemical localization of GUS activity in hypocotyls, 5-day-old seedlings grown in the dark and 7-day-old seedlings grown in the light were subjected to GUS staining. GUS assays were performed as described by Jefferson et al.^49^. The transgenic plants were incubated in GUS staining buffer (0.1 M phosphate-buffered saline (PBS), 0.5 mM potassium ferricyanide, 0.5 mM potassium ferrocyanide, 0.1% [v/v] Triton X-100, 2 mM X-Gluc) for 5–8 h at 37 °C.

### RNA extraction and quantitative real-time PCR analysis

Total RNA was extracted from *Arabidopsis* seedlings with an RNA purification kit (Bio Teke). RNase-free DNase Ι (TaKaRa) was used to remove genomic DNA and reverse transcription was performed with M-MLV reverse transcriptase (TaKaRa). Quantitative real-time PCR was carried out with an ABI 7500 PCR system (Applied Biosystems) using SYBR Premix Ex Taq (TaKaRa).

### Split-luciferase complementation assay

The coding sequences of *MREL57* and *MREL57*^*C486A*^ were cloned into *pCAMBIA-nLuc* vectors, and the coding sequences of *WDL7* and *WDL5* were cloned into *pCAMBIA-cLuc* vectors. The constructed plasmid vectors were transformed into Agrobacterium (strain GV3101) and then infiltrated into *Nicotiana benthamiana* leaves. LUC signal was detected by a cold charged-coupled camera (Nikon-L936; Andor Tech) after a 2-day cultivation in a growth room.

### Yeast two-hybrid assay

For yeast two-hybrid screening, a normalized universal *Arabidopsis* cDNA library (Cat. no. 630487; Clontech) was used. The yeast two-hybrid assay was performed according to the Yeast Protocols Handbook (Clontech). The coding sequences of *MREL57*, *MREL57N*, *MREL57C*, *JUL1*, *MBR2*, *TEAR1,* and *TEAR2* were cloned into *pGADT7* vector, and the coding sequences of *WDL7* and *WDL4* were cloned into *pGBKT7* vector. *pGBK-WDL3* was described previously^7^. The resulting vectors were co-transformed into yeast strain AH109. Transformed yeast cells were separately sprayed onto 2D synthetic dropout medium (-Trp/-Leu) and 4D selective medium (-Trp/-Leu/-His/-Ade) and incubated at 28 °C for 2–3 days.

### Semi-in vivo pull-down assay

For a semi-in vivo pull-down assay, MBP or MBP-WDL7-FLAG was used to pull-down MREL57-GFP from seedlings. Total proteins were extracted from 10-day-old *MREL57-GFP* transgenic seedlings with extraction buffer (50 mM Tris-HCl, pH 7.5; 150 mM NaCl; 10 mM MgCl_2_; 0.1% NP-40). MBP or MBP-WDL7-FLAG protein retained on the beads was incubated with the extracted proteins for 2 h at 4 °C and the beads were then washed three times with PBS buffer. The pulled down proteins were detected by immunoblot analysis using anti-GFP antibody (Roche 11814460001, 1/5000).

### In vitro ubiquitination assay

In vitro ubiquitination assays were performed according to the previous description^7,50^. Approximately 1 μg of purified MBP-MREL57-MYC or MBP was mixed with 100 ng wheat (*Triticum aestivum*) E1, 250 ng purified *Arabidopsis* E2 (AtUBC10), 5 μg ubiquitin (Boston Biochem, Cat. No. U-100AT), and 500 ng purified MBP-WDL7-FLAG in buffer containing 50 mM Tris-HCl (pH 7.4), 2 mM ATP, 5 mM MgCl_2_, 2 mM DTT, and 0.05 mM ZnCl_2_. After incubation at 30 °C for 3 h, the reactions were stopped with 5× sodium dodecyl sulfate sample buffer. The reaction products were analyzed by 6% sodium dodecyl sulphate polyacrylamide gel electrophoresis (SDS-PAGE) and immunoblotted using the appropriate antibodies. Self-ubiquitination of MBP-MREL57-MYC was detected with anti-MYC antibody (ABclonal AE010, 1/5000). Ubiquitinated MBP-WDL7-FLAG was detected with anti-FLAG antibody (ABclonal AE005, 1/5000).

### In vivo ubiquitination assay

An in vivo ubiquitination assay was performed as described previously^51^. Ten-day-old WDL7-GFP and WDL7-GFP/*mrel57-1* transgenic seedlings were treated with 1/2 MS liquid medium supplemented with 0 or 10 μM of ABA (Sigma) for 1 h in the presence of 50 μM MG132 (Sigma). Total proteins were extracted with extraction buffer and incubated for 2 h with anti-GFP mAb-magnetic agarose (MBL, Cat. No. D153-10). The beads were then washed three times with PBS buffer. The proteins were separated on 6% SDS-PAGE and WDL7-GFP ubiquitination was detected with anti-Ub antibody (Santa Cruz Biotechnology SC8017, 1/2000).

### Low-temperature and dilution assays

Low-temperature and dilution assays were performed as described previously^26,27^. His-WDL7-GFP protein (0 or 3 μM) was added to 20 μM rhodamine-labeled tubulin in PEM buffer (1 mM MgCl_2_, 1 mM EGTA, and 100 mM PIPES-KOH, pH 6.9) containing 1 mM GTP and the samples were then incubated at 35 °C for 40 min to allow tubulin assembly. For the low-temperature assay, the temperature was immediately decreased to 10 °C and maintained for 30 min. For the dilution assay, the assembled tubulin samples described above were diluted with 50× prewarmed PEM buffer containing His-WDL7-GFP protein (0 or 3 μM) and incubated at 35 °C for 60 min prior to fixation. The samples were fixed with 1% (v/v) glutaraldehyde for observation under confocal microscopy.

### Observation and quantification of cortical microtubules in cells

The rosette leaves of plants were incubated in opening solution (MES buffer) in a growth chamber for 2 h to open stomata completely. The leaves were then transferred to MES buffer containing different drugs for the indicated time periods. Cortical microtubules in pavement cells and guard cells were observed using a confocal laser scanning microscope (LSM 880, Carl Zeiss). YFP was excited at 488 nm and mCherry was excited at 561 nm. To preprocess the quantitative evaluations of microtubule density, maximum-intensity projections were constructed from serial optical sections. The images were then binarized by thresholding and skeletonized using the ImageJ menu “Process-Binary-Skeletonize”. The density of microtubules was defined as the occupancy of the GFP signal in guard cells^23^. To quantify the extent of microtubule bundling in guard cells, skewness of the intensity distribution of the microtubule pixels was measured as previously described^23,52^. Micrographs were analyzed with ImageJ using Higaki’s macro^23^.To evaluate microtubule orientation, the angle of each microtubule to the inner surface of the stomata was measured using ImageJ software^23^. All the analyses were carried out using the 8 bit raw scanning images.

### Reporting summary

Further information on research design is available in the Nature Research Reporting Summary linked to this article.

## Supplementary information

Supplementary Information

Reporting Summary

## Data Availability

Sequence data of the genes described in this article can be found in the *Arabidopsis* Genome Initiative under the following accession numbers: At5g24870, *MREL57*; At1g70950, *WDL7*; At3g23090, *WDL3*; At2g35880, *WDL4*; At4g32330, *WDL5*; At5g52310, *RD29A*; At5g52300, *RD29B*; At2g20580, *RPN1A*; At4g38630, *RPN10*; At4g33950, *OST1*; At4g26080, *ABI1*; At5g10650, *JUL1*; At4g34040, *MBR2*; At1g53190, *TEAR1*; At3g15070, *TEAR2*. The source data underlying Figs. 1b, d, e, 2a–g, 3b–g, 4b, f, g, 5c, f–j, 6b–h, 7b–I, and Supplementary Figs. 2, 4b–e, 5a–e, 8b, c, 10b, c, 11b, 12b, d, 14a–c, 15, 16a, c are provided in the Source Data file. All data supporting findings of this manuscript are available from the article and Supplementary Information files, or from the corresponding author upon request. Source data are provided with this paper.

## Source data

Source Data
