# Supplementary material for: The E3 ligase MREL57 modulates microtubule stability and stomatal closure in response to ABA
Source: Nat Commun. 2021 Apr 12;12:2181. doi: 10.1038/s41467-021-22455-y (PMC8041845; doi:10.1038/s41467-021-22455-y)
Supplement: Supplementary file 1 — Supplementary Information [file 41467_2021_22455_MOESM1_ESM.pdf]

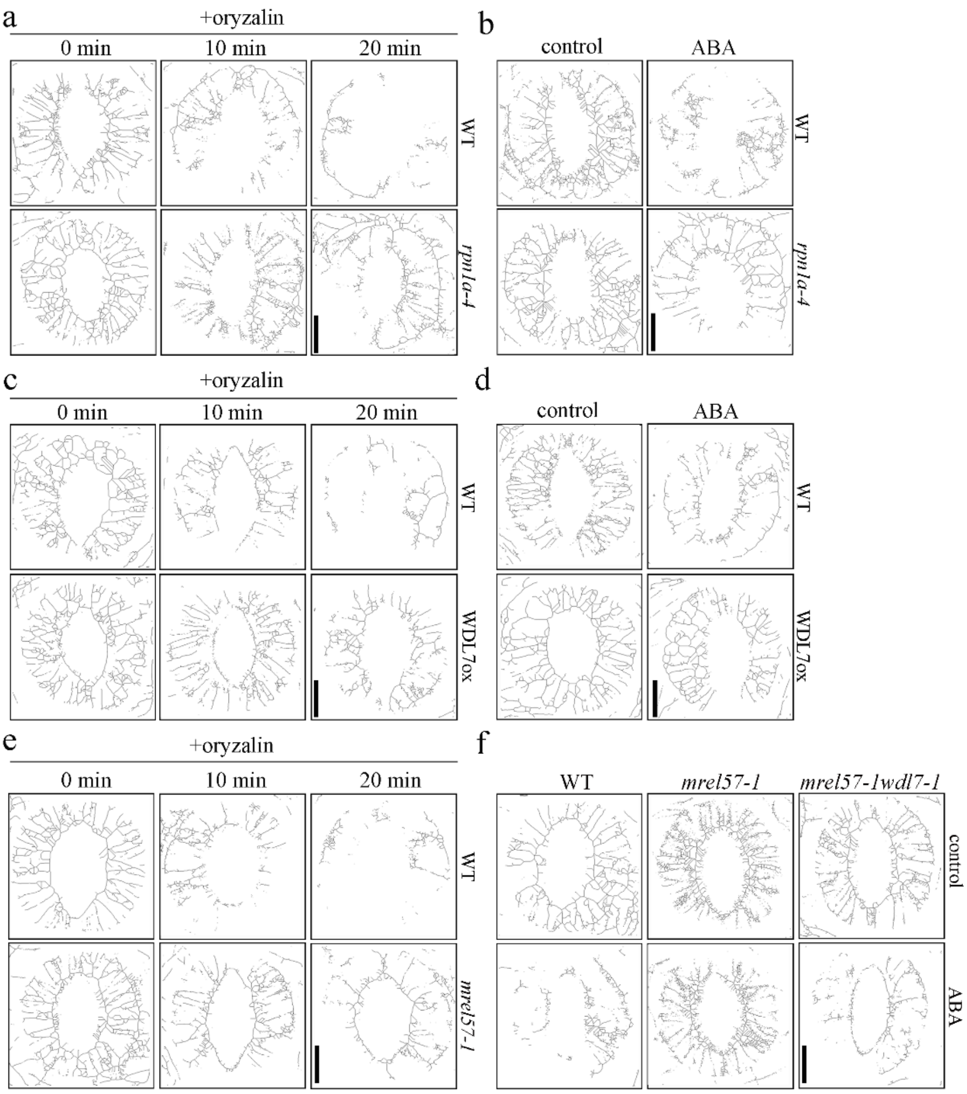

Supplementary Fig. 1 | The skeletonized images of microtubules in guard cells.
The obtained images of maximum intensity projection of serial optical sections were shown in Figures 1a, 1c, 4f, 4g, 6g, and 6h. Then the images were skeletonized using ImageJ software respectively. Scale bar = 5 μm.

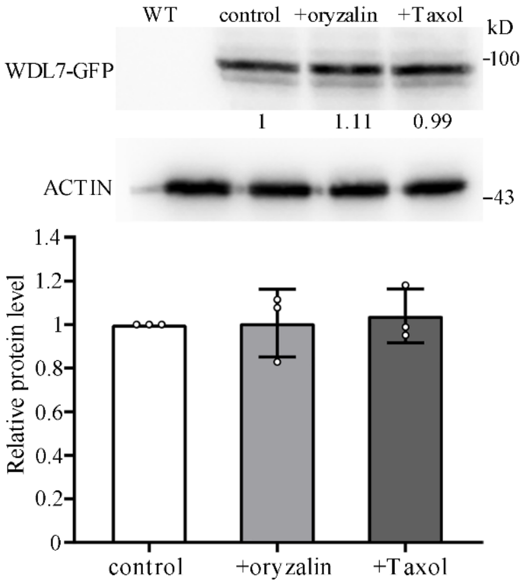

Supplementary Fig. 2 | Oryzalin and paclitaxel do not affect WDL7 stability. Ten-day-old *WDL7-GFP* transgenic seedlings were treated with mock buffer, 5 μM Oryzalin for 20 min, or 20 μM paclitaxel for 40 min. Total protein was extracted from the leaves. WDL7-GFP was detected with an anti-GFP antibody. Actin was used as a control. The WDL7-GFP protein level in the leaves of seedlings treated with mock buffer was set to 1 as a reference. Data represent the mean ± standard deviation (SD) values from three independent experiments.

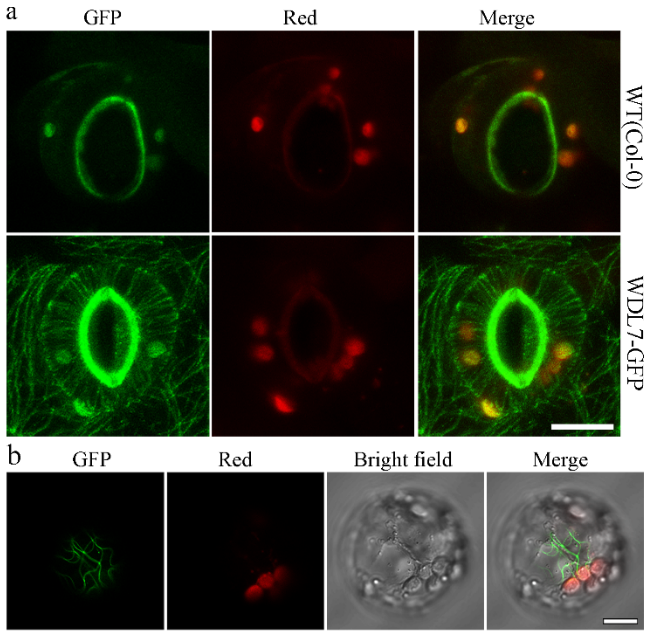

Supplementary Fig. 3 | WDL7 does not localize in chloroplasts.

a. Detached rosette leaves from wildtype and *WDL7-GFP* transgenic seedlings were incubated in opening buffer for 2 h. The red channel indicated the autofluorescence emitted by chloroplasts. Scale bar = 10 µm. Similar results were obtained for three independent experiments performed with a minimum of 10 cells each.

b. Subcellular localization of WDL7 in *Arabidopsis* protoplasts. *UBQ: WDL7-GFP* construct was transformed into *Arabidopsis* protoplasts and incubated for 12 h. Green and red represent GFP and chlorophyll autofluorescent signals. Scale bar = 10 µm.

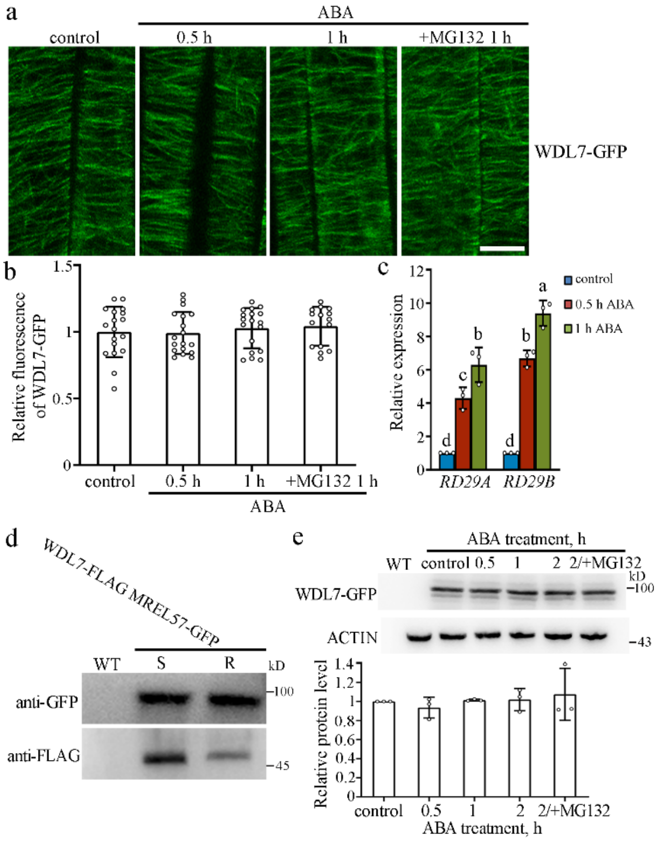

Supplementary Fig. 4 | WDL7 is not degraded in root in response to ABA.

a. 6-d-old WDL7-GFP transgenic plants were treated with 10 µM ABA or 10 µM ABA plus 50 µM MG132 for the indicated times. Bar = 50 µm.

b. The graph shows the relative fluorescence of WDL7-GFP in (a). Data represent the mean ± SD for three independent experiments with a minimum of 15 cells each.

c. Quantitative real-time PCR analysis of *RD29A* and *RD29B* RNA levels after treatment with 10 µM ABA for various durations in 6-d-old WDL7-GFP transgenic plants. *UBQ11* was used as the reference gene. Gene expression levels in the seedlings treated with mock buffer were set to 1. The data represent the mean ± SD for three independent experiments. Different letters represent significant differences at $p < 0.01$ (one-way ANOVA).

d. The co-IP assay shows the interactions of MREL57 and WDL7 in the shoots and roots. Total protein was extracted from the shoots and roots of ten-day-old *35S: WDL7-FLAG MREL57-GFP* transgenic seedlings. The protein samples were immunoprecipitated with GFP beads. MREL57-GFP was detected with an anti-GFP antibody, and WDL7-FLAG was detected with an anti-FLAG antibody. WT seedlings were used as a control. The experiment repeated three times with similar results.

e. Ten-day-old *WDL7-GFP* transgenic seedlings were treated with mock buffer, 10 µM ABA, 10 µM ABA plus 50 µM MG132 for indicated times and then total proteins were extracted from the root. WDL7-GFP was detected with anti-GFP antibody. Actin was used as a control. The graph shows the quantitative analysis of protein levels. The protein level of WDL7-GFP treated with mock buffer was set to 1 as a reference for calculating relative protein levels of various time points. Data represent the mean ± SD for three independent experiments.

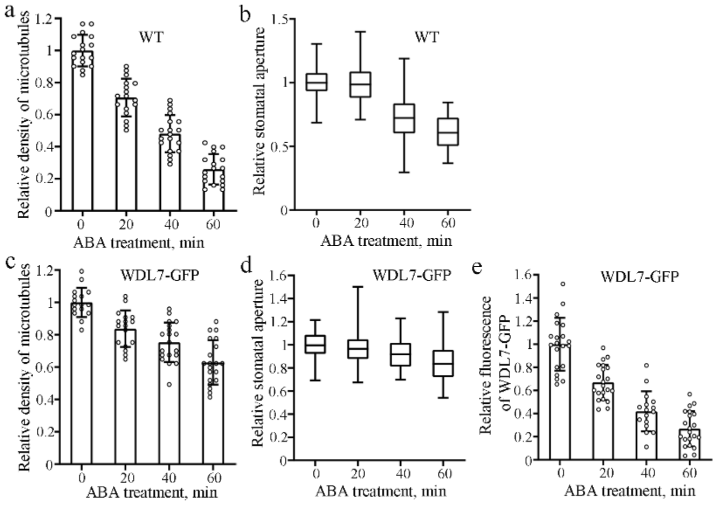

Supplementary Fig. 5 | In vivo time-course analysis of WDL7 degradation, microtubule disassembly, and stomatal closure.

Detached rosette leaves from WT and *WDL7-GFP* transgenic seedlings on an *mCherry-tubulin* background were treated with 10 μM ABA for 20, 40, or 60 min.

a and c. The graphs show the relative microtubule densities in the guard cells from WT (a) or *WDL7-GFP* (c) transgenic seedlings. (a) n = 17 for sample 0 min, n = 17 for sample 20 min, n = 19 for sample 40 min, n = 18 for sample 60 min. (c) n = 16 for sample 0 min, n = 17 for sample 20 min, n = 19 for sample 40 min, n = 20 for sample 60 min. Data represent mean ± standard deviation (SD) values from three independent experiments.

b and d. The graphs show the relative stomatal aperture of WT (b) or *WDL7-GFP* (d) transgenic seedlings. The box and whiskers plots represent minimum and maximum values. The line in the box indicates the median value and the boundaries demonstrate the 25th percentile (upper) and the 75th percentile (lower). (b) n = 130 for sample 0 min, n = 223 for sample 20 min, n = 290 for sample 40 min, n = 188 for sample 60 min. (d) n = 143 for sample 0 min, n = 180 for sample 20 min, n = 124 for sample 40 min, n = 150 for sample 60 min. The experiment was repeated three times as different biological replicates with a minimum of 100 stomatal pores.

e. The graph shows the relative fluorescence in guard cells from WDL7-GFP transgenic seedlings. Data represent mean ± standard deviation (SD) values from three independent experiments. n = 20 for sample 0 min, n = 21 for sample 20 min, n = 16 for sample 40 min, n = 21 for sample 60 min.

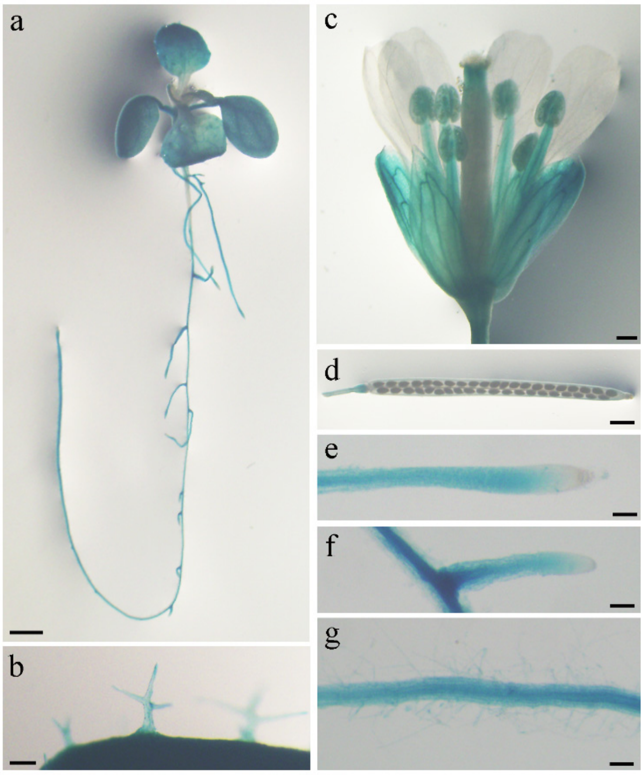

Supplementary Fig. 6| Expression patterns of *WDL7* by GUS staining.
The *WDL7* gene expressed in the young seedling (**a**), trichome (**b**), flower (**c**), silique (**d**), primary root (**e**), lateral root **(f)** and root hair (**g**). Scale bars = 1 mm in (a) and (d); 0.1 mm in (b), (e), (f) and (g); 0.2 mm in (c). The experiment repeated three times with similar results.

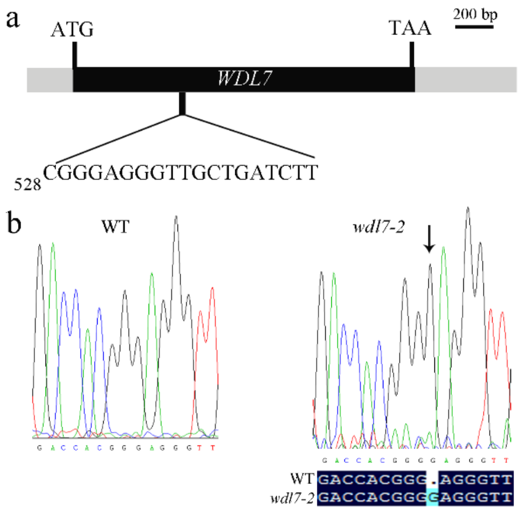

Supplementary Fig. 7 | Generation of *wdl7* loss-of-function mutant.

a. Diagram showing the target sites of the CRISPR/Cas9 system for *WDL7*.

b. The *wdl7-2* mutant was identified by sequencing and the mutation site in *WDL7* was indicated by arrow.

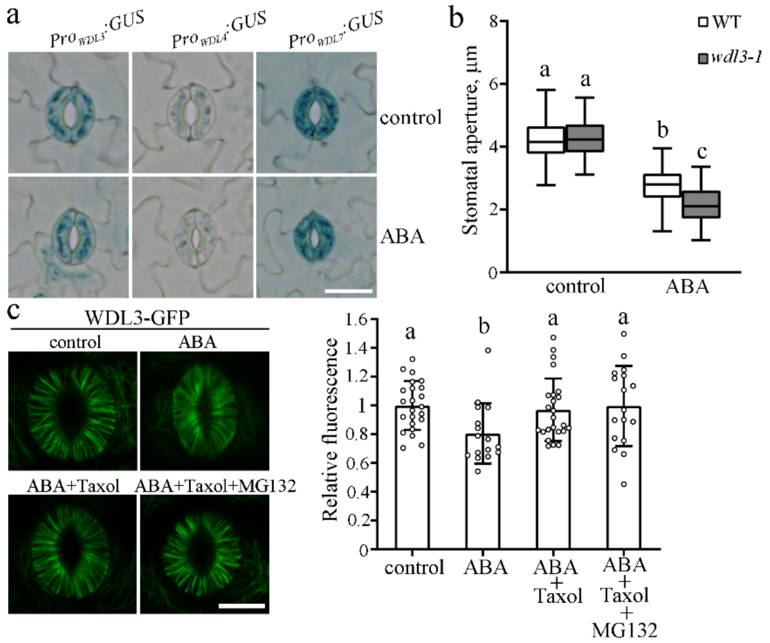

Supplementary Fig. 8 | WDL3 and WDL4 do not participate in UPS-mediated ABA-regulated stomatal closure.

a. Detached rosette leaves from *Pro_{WDL3}: GUS*, *Pro_{WDL4}: GUS* and *Pro_{WDL7}: GUS* transgenic seedlings were incubated in opening buffer for 2 h and then treated with 10 μM ABA for 2 h. The epidermal strips were peeled from the rosette leaves, and GUS staining was performed. The experiment repeated three times with similar results. Scale bar = 20 μm.

b. Detached rosette leaves from WT and *wdl3-1* seedlings were incubated in opening buffer for 2 h and then treated with 10 μM ABA for 2 h. The box and whiskers plots represent minimum and maximum values. The line in the box indicates the median value and the boundaries demonstrate the 25th percentile (upper) and the 75th percentile (lower). Different letters represent significant differences at $p < 0.01$ (one-way ANOVA). The experiment was repeated three times as different biological replicates with a minimum of 100 stomatal pores.

c. Detached rosette leaves from *WDL3-GFP* transgenic seedlings were incubated in opening buffer for 2 h and then treated with 10 μM ABA, 10 μM ABA plus 20 μM Taxol, 10 μM ABA plus 20 μM Taxol and 50 μM MG132 for 40 min. Scale bar = 10 μm. The graphs show the relative fluorescence of WDL3-GFP. Data represent mean ± standard deviation (SD) values from three independent experiments with a minimum of 15 cells each. Different letters represent significant differences at $p < 0.01$ (one-way ANOVA).

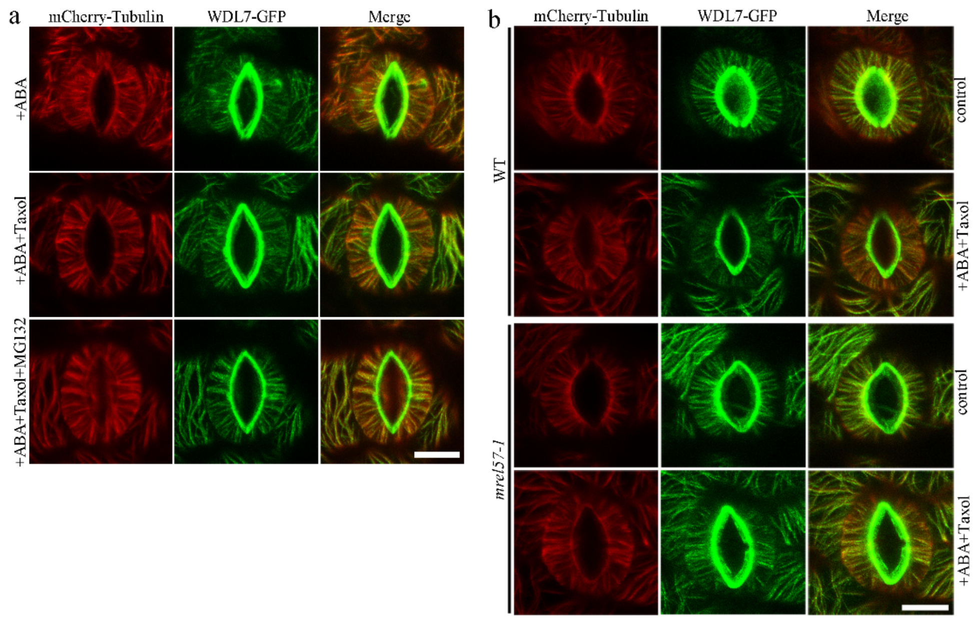

Supplementary Fig. 9 | WDL7-GFP colocalizes with cortical microtubules in guard cells in response to ABA.

a. Detached rosette leaves of seedlings from *WDL7-GFP* transgenic plants on an *mCherry-tubulin* background were incubated in opening buffer for 2 h and then treated with 10 µM ABA, 10 µM ABA plus 20 µM Taxol, 10 µM ABA plus 20 µM Taxol and 50 µM MG132 for 40 min. Scale bar = 10 µm. Similar results were obtained for three independent experiments performed with a minimum of 10 cells each.

b. Detached rosette leaves from *WDL7-GFP mCherry-tubulin* transgenic seedlings on a WT or *mrel57-1* mutant background were incubated in opening buffer for 2 h and then treated with 10 µM ABA plus 20 µM Taxol for 40 min. Scale bar = 10 µm. Similar results were obtained from three independent experiments (n $\geq$ 10 cells per experiment).

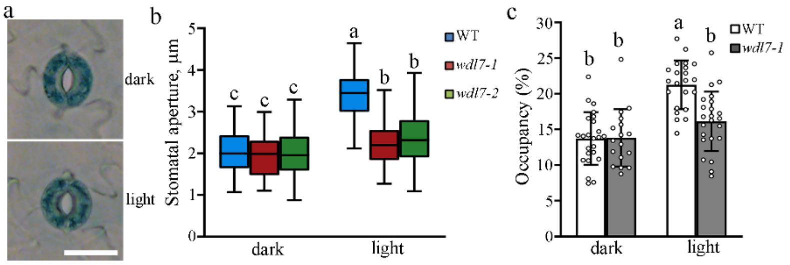

Supplementary Fig. 10 | WDL7 participates in light-induced stomatal opening.

a. Detached rosette leaves from *Pro*$_{WDL7}$*: GUS* transgenic seedlings were incubated in the dark for 4 h and then transferred to the light for 1 h. The epidermal strips were peeled from the rosette leaves and GUS staining was performed. Scale bar = 20 µm.

b. Light-induced stomatal opening in the wildtype, *wdl7-1* and *wdl7-2*. Detached rosette leaves from WT, *wdl7-1* and *wdl7-2* were preclosed in darkness for 4 h and then incubated in the light for 1 h. The box and whiskers plots represent minimum and maximum values. The line in the box indicates the median value and the boundaries demonstrate the 25th percentile (upper) and the 75th percentile (lower). Different letters represent significant differences at $p < 0.01$ (one-way ANOVA). The experiment was repeated three times as different biological replicates with a minimum of 100 stomatal pores.

c. Detached rosette leaves from WT and *wdl7-1* transgenic seedlings on a *YFP-tubulin* background were incubated in the dark for 4 h and then transferred to the light for 1 h. The graph shows the densities of the microtubules. Data represent the mean ± SD for three independent experiments with a minimum of 15 cells each. Different letters represent significant differences at $p < 0.01$ (one-way ANOVA).

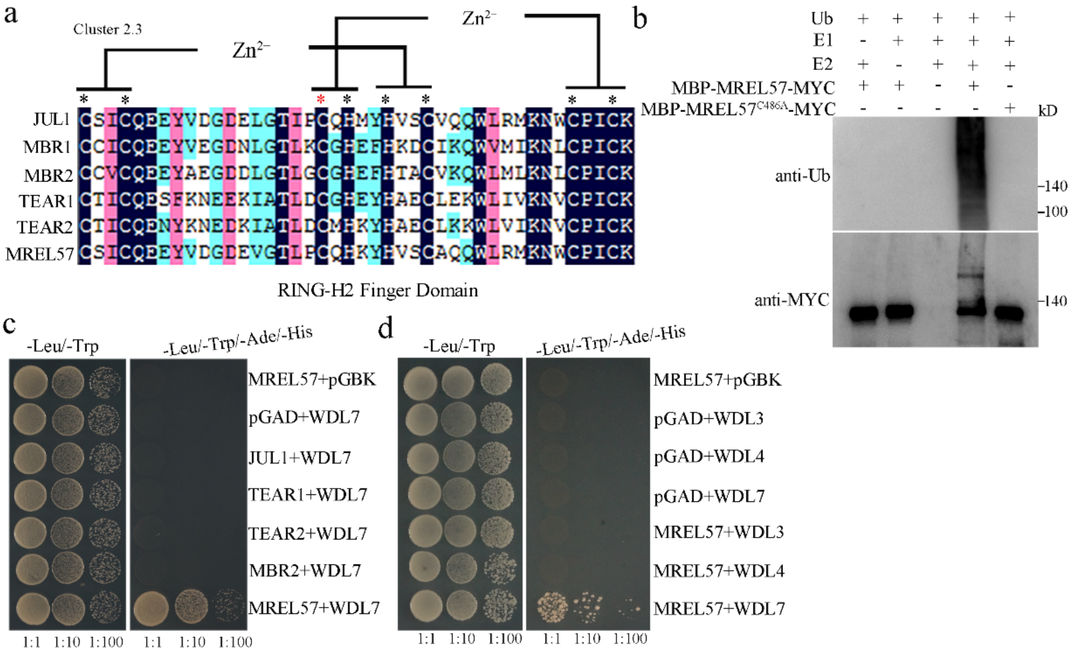

Supplementary Fig. 11 | Ubiquitin ligase activity of MREL57.

a. Sequence alignment of the RING-H2 domain at the C-terminal end of MREL57 and five known E3 ligases in cluster 2.3. The conserved metal-ligand residues of cysteine or histidine are marked with asterisks, and the red asterisk marks the amino acid to be mutated.

b. The ubiquitination reaction contains E1, E2 (*Arabidopsis* UBC10), ubiquitin (Ub), MBP-MREL57-MYC, or MBP-MREL57^{C486A}-MYC. Polyubiquitin chains were observed using immunoblotting with anti-Ub and anti-MYC antibodies. The experiment repeated three times with similar results.

c. The yeast two-hybrid assay showed that WDL7 interacted with MREL57 but not with JUL1, MBR2, TEAR1, or TEAR2. Empty vectors were used as negative controls. The experiment repeated three times with similar results.

d. The yeast two-hybrid assay showed that MREL57 interacted with WDL7 but not with WDL3 or WDL4. Empty vectors were used as negative controls. The experiment repeated three times with similar results.

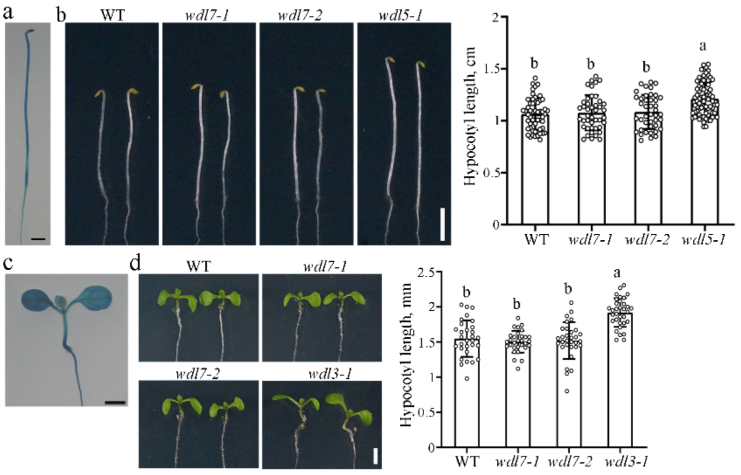

Supplementary Fig. 12 | WDL7 may not be involved in hypocotyl elongation.

a. Histochemical GUS staining in 5-day-old Pro_{WDL7}: *GUS* transgenic seedlings grown in the dark. Scale bar = 1 mm.

b. WT and *wdl7* and *wdl5-1* mutants were grown on MS medium in the dark for five days. The *wdl5-1* mutant was used as a positive control. Scale bar = 3 mm. The graph shows the average hypocotyl length in (b). Data represent the mean ± SD for three independent experiments with a minimum of 40 seedlings each. Different letters represent significant differences at $p < 0.01$ (one-way ANOVA).

c. Histochemical staining of GUS in 7-day-old Pro_{WDL7}: *GUS* transgenic seedlings grown in the light. Scale bar = 1 mm.

d. WT and *wdl7* and *wdl3-1* mutants were grown on 1/2 MS medium in the light for seven days. The *wdl3-1* mutant was used as a positive control. Scale bar = 2 mm. The graph shows the average hypocotyl length in (d). Data represent the mean ± SD for three independent experiments with a minimum of 30 seedlings each. Different letters represent significant differences at $p < 0.01$ (one-way ANOVA).

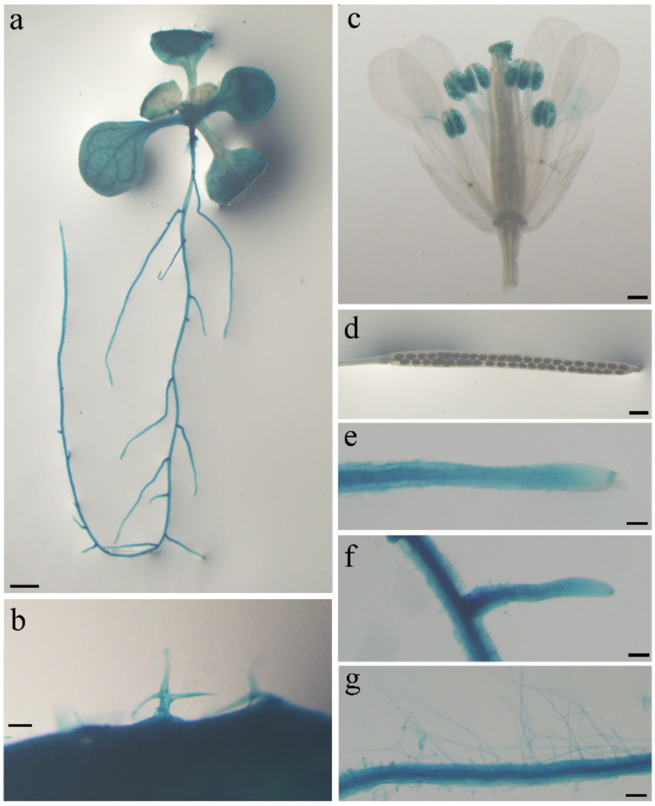

Supplementary Fig. 13 | Expression patterns of *MREL57* by GUS staining.
Expression patterns of *Pro$_{MREL57}$: GUS* in the young seedling (**a**), trichome (**b**), flower (**c**), silique (**d**), primary root (**e**), lateral root (**f**) and root hair (**g**). Scale bars = 1 mm in (a) and (d); 0.1 mm in (b), (e), (f) and (g); 0.2 mm in (c). The experiment repeated three times with similar results.

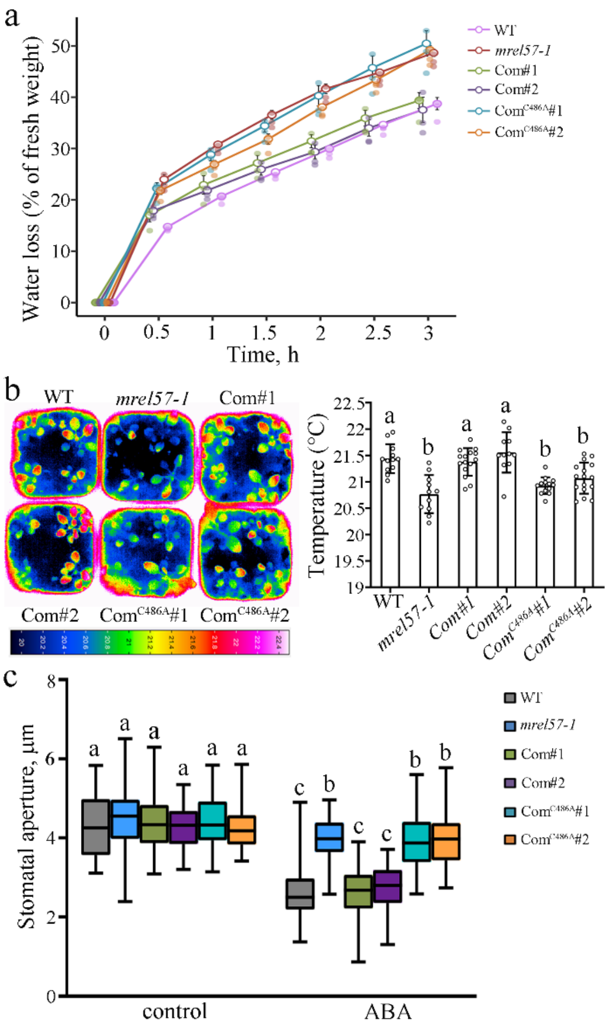

Supplementary Fig. 14 | Stomatal phenotypes of *mrel57-1* mutant are completely restored by *Pro$_{MREL57}$: MREL57-GFP,* but not *Pro$_{MREL57}$: MREL57^{C486A}-GFP*, in response to ABA.

a. Fresh weights of the detached leaves of seedlings from WT, *mrel57-1*, Com#1, Com#2, Com^{C486A} #1, and Com^{C486A} #2 were measured at the indicated times. The experiment was repeated three times with independent treatments.

b. Infrared thermography of WT, *mrel57-1*, Com#1, Com#2, Com^{C486A}#1, and Com^{C486A}#2. The images of 4-week-old plants in soil were taken using an infrared camera. Leaf temperature was measured using infrared camera software. Data represent mean ± standard deviation (SD) values. Different letters represent significant differences at $p < 0.01$ (one-way ANOVA). The experiment was repeated three times as different biological replicates.

c. Detached rosette leaves of seedlings from WT, *mrel57-1*, Com#1, Com#2, Com^{C486A} #1, and Com^{C486A} #2 were incubated in opening buffer for 2 h and then treated with 10 µM ABA for 2 h. The box and whiskers plots represent minimum and maximum values. The line in the box indicates the median value and the boundaries demonstrate the 25th percentile (upper) and the 75th percentile (lower). Different letters represent significant differences at $p < 0.01$ (one-way ANOVA). The experiment was repeated three times as different biological replicates with a minimum of 100 stomatal pores.

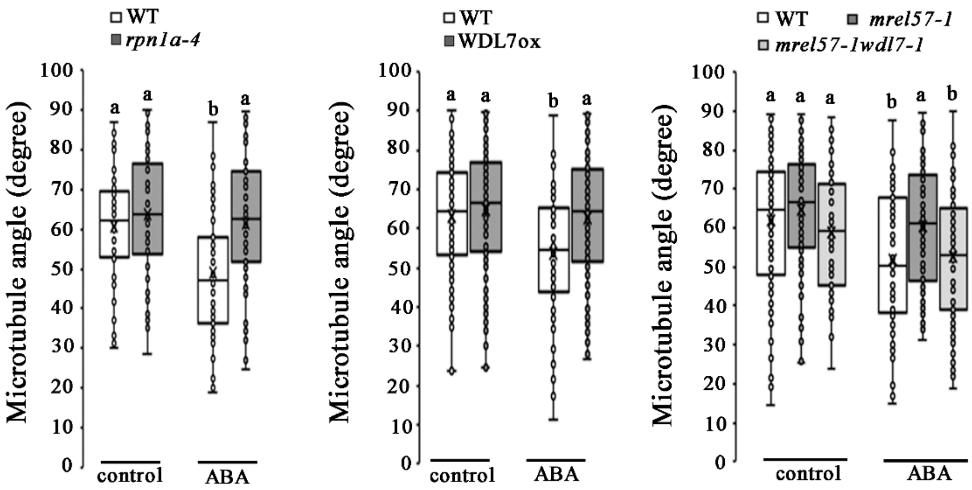

Supplementary Fig. 15 | Quantification of cortical microtubule orientation in response to ABA.

The orientation of the cortical microtubules (shown in Figures 1c, 4g, and 6h) was determined by measuring the angles between the cortical microtubules and the nearest stomatal pore edge (metrics for microtubule orientation). The box and whiskers plots represent minimum and maximum values. The cross indicates the mean microtubule angles, and the segments inside the box indicate the median microtubule angles (n > 90). The experiment was repeated three times with similar results. Different letters represent significant differences at $p < 0.01$ (one-way ANOVA).

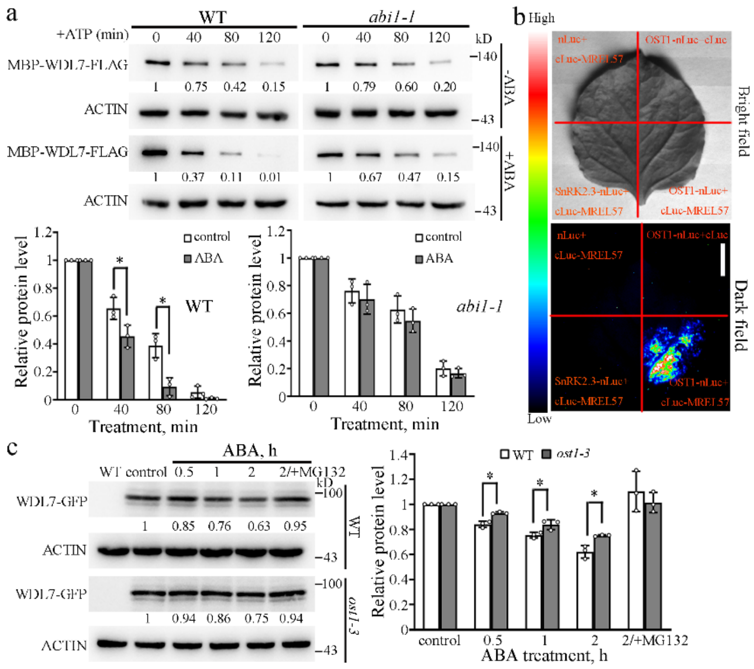

Supplementary Fig. 16 | ABA signaling mediates the degradation of WDL7.

a. Degradation of WDL7 was clearly decreased in *abi1-1* mutant in a cell-free degradation assay. Purified MBP-WDL7-FLAG was incubated with equal amounts of total proteins from 10-day-old WT and *abi1-1* seedlings. MBP-WDL7-FLAG was detected with anti-FLAG antibody. Actin was used as a control. The graphs show the quantitative analysis of protein levels. The WDL7 protein level at 0 h was set to 1 as a reference for calculating the relative protein levels at the various time points. Data represent the mean ± SD for three independent experiments. Two-tailed Student's *t* test, $*p < 0.05$.

b. Split-luciferase complementation assay to analyze the interaction between MREL57 and OST1 in *N. benthamiana* leaves. SnRK2.3 was used as a negative control. The pseudo-color bar shows the range of luminescence signals. Scale bar = 1 cm.

c. Degradation of WDL7 was significantly decreased in *ost1-3* mutant. Ten-day-old *WDL7-GFP* transgenic seedlings on the wild type or the *ost1-3* mutant background were treated with mock buffer, 10 μM ABA, or 10 μM ABA plus 50 μM MG132 for indicated times and then total proteins were extracted from the leaves. WDL7 was detected with anti-GFP antibody and actin was used as a control. The WDL7 protein level treated with mock buffer was set to 1 as a reference for calculating the relative protein levels. Data represent the mean ± SD for three independent experiments. Two-tailed Student's *t* test, $*p < 0.05$.

Supplementary Table 1. List of primer sequences used in this study.

Primers for quantitative real-time PCR

Primer name	Primer sequence (5'-3')
MREL57-F	TAATGAAGACTCACCCACAA
MREL57-R	CTTCCTGCCTCTGTCACAAC
WDL7-F	CAGAAA CAAACCAAGTTCAGGCC
WDL7-R	TCTGAATCTTTAGCCGCACTCTG
UBQ11-F	GCAGATTTTCGTTAAAACC
UBQ11-R	CCAAAGTTCTGCCGTCC
RD29A-F	CAGAGGAACCACCACTCAAC
RD29A-R	TTTCCTCCGATGCTGGAACA
RD29B-F	TGACTCCGGTTTACGAAAAAGT
RD29B-R	GTCTCCTTCACTCCACTTCCAC

Primers for constructs in plant transformation

Primer name	Primer sequence (5'-3')
WDL7-BsF	ATATATGGTCTCGATTGGGCGGTGGTGACCACGGGAGTT
WDL7-F0	TGGGCGGTGGTGACCACGGGAGTTTTAGAGCTAGAAATAGC
WDL7-R0	AACCGGGAGGGTTGCTGATCTTCAATCTCTTAGTCGACTCTAC
WDL7-BsR	ATTATTGGTCTCGAAACCGGGAGGGTTGCTGATCTTC
MREL57-F	ACTAGTATGGATGGATTTAAGGGTA
MREL57-R	GGTACCGGCCAACAATACCTCTGCAACTCCA
WDL7-F	GGATCCATGGCAGGAGAGATTCAGGACCCGT
WDL7-R	GGTACCGTTTCTGCTTCTTCGA
WDL7-native-Pro-F	GTCGACTCATTTCCCTTTAGATTTTCTTG
WDL7-native-Pro-R	GGATCCAATTGATCTCAAGGAAAATAAG
MREL57-native-Pro-F	GTCGACTTCTTTGTTTCTTCTCTTCAC
MREL57-native-Pro-R	GGATCCACATAAAACACACCCTCAAACACTG
WDL4-native-Pro-F	GTCGACTCACTCACTTTAGTCTCTTAGAGCTG
WDL4-native-Pro-R	GGATCCTCAGGGACAACTTCCAAATCC
MREL57-F	GGATCCATGGATGGATTTAAGGGTA
MREL57-R	GGTACCTGAAAATGGATGTGGCTGAGA

Primers for genotyping

Primer name	Primer sequence (5'-3')
wdl7-1-LP	AGAATGAAGGCATTGGTGTTG
wdl7-1-RP	ATCTTGGACCTCCTGAGATGC
mrel57-1-LP	CGAACCAATACAAGGTGAAAAC
mrel57-1-RP	GTAGAACAGGACGTGCTGGAG
mrel57-2-LP	TTCTCTTCACGTCGTCTTTCC
mrel57-2-RP	GCTCATTCGATTTACACGGAG
rpn1a-4-LP	AATATGTCGAAATCCTCTTCAAACG
rpn1a-4-RP	ACCATCAGAATTCAGAAGGATAAAC
ost1-3-LP	CATATCTTTAGACGAGGGGCC
ost1-3-RP	GTGAGTGGTCCAATGGATTTG
LBb1.3	ATTTTGCCGATTTCGGAAC

Primers for yeast two-hybrid and split-luciferase complementation assay

Primer name	Primer sequence (5'-3')
MREL57-F	GAATTCATGGATGGATTTAAGGGTA
MREL57-R	GGATCCGGCCAACAATACCTCTGCAACTCCA
MREL57 N-F	GAATTCATGGATGGATTTAAGGGTA
MREL57 N-R	GGATCCGGCCAACAATACCTCTGCAACTCCA
MREL57 C-F	GAATTCCTGGAAAGGATTGAGCAAGACG
MREL57 C-R	GGATCCGGCCAACAATACCTCTGCAACTCCA
WDL7-F	CATATGATGGCAGGAGAGATTCAGGAC
WDL7-R	GGATCCCTAGTTTCTGCTTCTTCGAGC
WDL4-F	CATATGATGGCGTCCGAGGATTTGAATATTG
WDL4-R	GTCGACTCAGCCTCCCACTACAACCT
MREL57-F	GGATCCATGGATGGATTTAAGGGTA
MREL57-R	GTCGACTGAAAATGGATGTGGCTGAGA
WDL7-F	GGATCCATGGCAGGAGAGATTCAGGACCCGT
WDL7-R	GTCGACCTAGTTTCTGCTTCTTCGA
WDL5-F	GGATCCATGGACCCTGAGAGTATCATGGC
WDL5-R	GTCGACTTAATGCTCAACAGCAACCGC
MBR2-F	GAATTCATGCAAGGTCCACGAAGCACT
MBR2-R	CCCGGGTTATGTCGATAAAGCCACAGTCT
TEAR1-F	CATATGATGGGACAAAGAAATAGGAATGTTG

TEAR1-R	GAATTCCTATACCTTTCTCTTCTCCATGACC
TEAR2-F	CCCGGGTATGCGACAAAGAAATATGATGACTG
TEAR2-R	CTCGAGTCATCTACTACTTAACCTTAGCT
JUL1-F	GAATTCATGGATGGATGTGCTGGTAAACGAT
JUL1-R	GGATCCCTAAATCGACTTCTCTTCTTCCG

Primers for expressing protein in vitro

Primer name	Primer sequence (5'-3')
WDL7-F	GGATCCATGGCAGGAGAGATTCAGGACCCGT
WDL7-R	GTCGACCTACTTATCGTCGTCATCCTTGTAATCGTTTCT GCTTCTTCGA
MREL57-F	GGATCCATGGATGGATTTAAGGGTA
MREL57-R	GTCGAC TTATACCGAGTTCAAGTCCTCT
MREL57^{C486A}-F	GTAGGGACTTTGCCTGCA CAACATA
MREL57^{C486A}-R	TTCATCTCCATCAACATACTCTTCC
WDL7-F	GGTACCATGGCAGGAGAGATTCAGGACCCGT
WDL7-R	GGATCCGTTTCTGCTTCTTCGA
